# Sub-Chronic Neuropathological and Biochemical Changes in Mouse Visual System after Repetitive Mild Traumatic Brain Injury

**DOI:** 10.1371/journal.pone.0153608

**Published:** 2016-04-18

**Authors:** Radouil Tzekov, Clint Dawson, Megan Orlando, Benoit Mouzon, Jon Reed, James Evans, Gogce Crynen, Michael Mullan, Fiona Crawford

**Affiliations:** 1 The Roskamp Institute, Sarasota, FL, United States of America; 2 Department of Ophthalmology, University of South Florida, Tampa, FL, United States of America; 3 James A. Haley Veteran’s Administration, Tampa, FL, United States of America; Hanson Institute, AUSTRALIA

## Abstract

Repetitive mild traumatic brain injury (r-mTBI) results in neuropathological and biochemical consequences in the human visual system. Using a recently developed mouse model of r-mTBI, with control mice receiving repetitive anesthesia alone (r-sham) we assessed the effects on the retina and optic nerve using histology, immunohistochemistry, proteomic and lipidomic analyses at 3 weeks post injury. Retina tissue was used to determine retinal ganglion cell (RGC) number, while optic nerve tissue was examined for cellularity, myelin content, protein and lipid changes. Increased cellularity and areas of demyelination were clearly detectable in optic nerves in r-mTBI, but not in r-sham. These changes were accompanied by a ~25% decrease in the total number of Brn3a-positive RGCs. Proteomic analysis of the optic nerves demonstrated various changes consistent with a negative effect of r-mTBI on major cellular processes like depolymerization of microtubules, disassembly of filaments and loss of neurons, manifested by decrease of several proteins, including neurofilaments (NEFH, NEFM, NEFL), tubulin (TUBB2A, TUBA4A), microtubule-associated proteins (MAP1A, MAP1B), collagen (COL6A1, COL6A3) and increased expression of other proteins, including heat shock proteins (HSP90B1, HSPB1), APOE and cathepsin D. Lipidomic analysis showed quantitative changes in a number of phospholipid species, including a significant increase in the total amount of lysophosphatidylcholine (LPC), including the molecular species 16:0, a known demyelinating agent. The overall amount of some ether phospholipids, like ether LPC, ether phosphatidylcholine and ether lysophosphatidylethanolamine were also increased, while the majority of individual molecular species of ester phospholipids, like phosphatidylcholine and phosphatidylethanolamine, were decreased. Results from the biochemical analysis correlate well with changes detected by histological and immunohistochemical methods and indicate the involvement of several important molecular pathways. This will allow future identification of therapeutic targets for improving the visual consequences of r-mTBI.

## Introduction

Traumatic brain injury (TBI) is a major cause of death and disability worldwide, including in the US, where annual incidence is approximately 1.7 million [1]. TBI severity is commonly described as mild, moderate, or severe based on a number of factors, but concussion, or mild TBI (mTBI), is the most common form of TBI, accounting for up to 75% of all brain injuries occurring annually in the US [1]. It is estimated that mTBI re-occurs in at least 6–7% of the cases as repeated mTBI (r-mTBI) [2, 3].

Ocular and vision damage have been reported previously as a consequence of TBI, including mTBI, as part of the post-concussion syndrome, including symptoms of photosensitivity and blurred vision [4–7]. The mechanisms leading to these negative impacts on the visual system are still unclear and are the subject of intensive research by many investigators.

We have developed a mouse model to enable characterization of the consequences of r-mTBI in closed head injury [8, 9]. Extensive neurobehavioral and neuropathological analyses of this model have demonstrated the deleterious effects of r-mTBI on brain morphology and cognitive function. Furthermore, we described negative consequences for the visual system at 10 to 13 weeks post injury, such as decreased optic nerve diameters, increased cellularity and areas of demyelination in optic nerves in r-mTBI versus control (repeated sham, or r-sham) mice [10]. There were also concomitant areas of decreased cellularity in the retinal ganglion cell (RGC) layer and an approximately 67% decrease in the total number of Brn-3a-positive RGCs in retinal flatmounts. In addition, thinning of the inner retina and an effect on the visual function manifested by a decrease in the amplitude of the photopic negative response of the electroretinogram were observed.

The detailed characterization of the retinal and optic nerve changes at 10–13 weeks post injury indicated that significant pathologic changes may be present at earlier time points, thus similar characterization at an earlier time point was suggested to provide a better estimate for the rate of development of the pathological process and a potential therapeutic window. Furthermore, it was undetermined if it would be possible to do both proteomic and lipidomic analysis on a single mouse optic nerve specimen using the contralateral optic nerve for histology. If feasible, this option would allow a reduction in the number of animals in future studies.

The purpose of this study was to expand upon the findings of the previous study and to use this model to characterize the effects of r-mTBI at an earlier time point (3 weeks post injury) using neuropathological and biochemical methods.

## Materials and Methods

### Administration of TBI

All procedures were carried out according to the Association for Research in Vision and Ophthalmology statement for the Use of Animals in Ophthalmic and Vision Research, the Association for Assessment and Accreditation of Laboratory Animal Care and were approved by the local Institutional Animal Care and Use Committee (Roskamp Institute IACUC Protocol #56).

Adult C57BL/6 male mice (aged 8–10 weeks) were randomly assigned to either r-mTBI or repetitive sham (r-sham; anesthesia only) groups. Five consecutive hits with an inter-concussion interval of 48 hours were applied, according to an established method [8–10]. Briefly, mice were anesthetized with an inhalation isoflurane anesthesia and placed on a heating pad with the head fixed in a stereotaxic frame. The skin around the impact area was shaved but remained intact. The injury was triggered using an electromagnetic controlled impact device with a 5-mm blunt metal impactor tip at a strike velocity of 5 m per second, strike depth of 1.0 mm, and dwell time of 200 msec. The location of the impact on the skull was central, equally distant from both eyes. After the injury, each mouse was placed on heating pad for recovery and once the effects of anesthesia wore off, was placed beck in its home cage and monitored for any signs of discomfort or pain. Sham mice received anesthesia of equal duration to the injured mice and at the same intervals. Cohorts of mice were euthanatized 3 weeks after r-mTBI (n = 5) or r-sham (n = 5), and the eyes and optic nerves (one optic nerve per animal) examined histologically. In addition, naive C57BL/6 mice (both male and female) aged 6 to 9 months (n = 7) were obtained from Jackson Laboratories (Bar Harbor, ME) and were used as a reference group for histology.

### Neuropathological analysis

Tissue preparation was carried out as described [10]. Briefly, animals were deeply anesthetized and perfused with heparinized PBS. The eyes and optic nerves were extracted separately by cutting the optic nerve approximately 0.5 mm from the posterior pole of the eyeball. Low-temperature cautery was used to mark the 12-o’clock position on each cornea and both eye and optic nerve specimens were fixed in 10% neutral buffered formalin (BBC Biochemical, Everett, WA) at 4°C for 24 hours and then transferred into 80% ethanol for long-term storage. Samples were processed for paraffin embedding using a standard protocol and embedded in paraffin in such a way as to ensure a cut through the vertical meridian of the eyeball and a longitudinal cut through the optic nerve.

Paraffin blocks were sectioned at a thickness of 4 microns and mounted on positively charged glass slides (Erie Scientific, Portsmouth, NH).

Hematoxylin and eosin (H&E) staining was applied to the optic nerves and eye cross sections. In addition, Luxol fast blue (LFB) staining with cresyl violet (CV) as a counterstain was applied to the optic nerve cross sections.

#### Retinal ganglion cell counting

**Fig 1:**
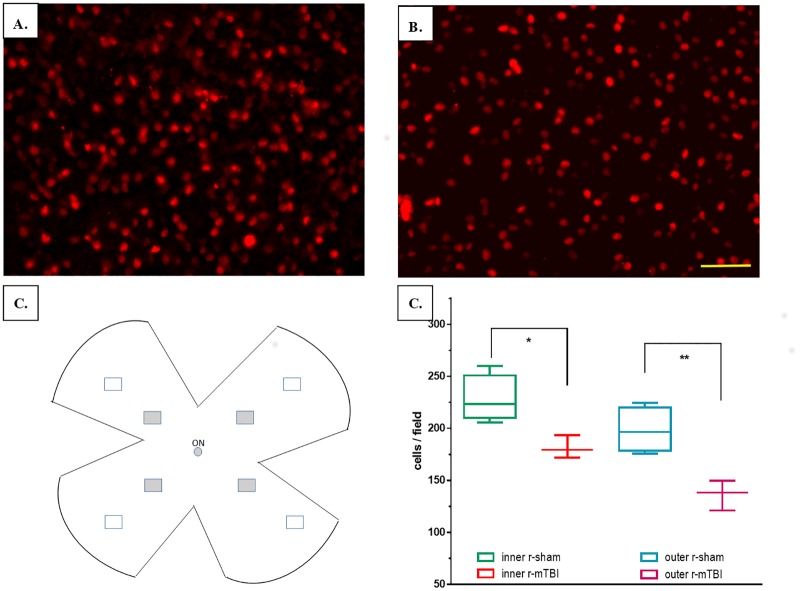
Representative images of retina flatmounts and results from RGC counts. Representative images from the upper retina of a mouse after repetitive anesthesia alone (r-sham) (A) and r-mTBI (B). Retinal ganglion cells are labeled with anti-Brn-3a antibody. Scale bar = 50 μm. (C) Drawing depicting the retinal samples used to calculate the cell density; grey filled rectangles indicate the position of inner areas, while unfilled rectangles indicate the position of the outer retinal areas; ON—optic nerve. (D) Comparison between the average cells/field (cell densities) in the inner and outer areas.

Whole-mount retinal preparations from mice at 3 weeks after injury (n = 4, r-sham; n = 3, r-mTBI) were carried out as described [10] based on a slight modification of a published method [11]. Briefly, mice were deeply anesthetized, and the vertical orientation of the eye was marked by low-temperature cautery. Retinas were dissected and prepared as flattened whole mounts by making 4 radial cuts, postfixed for an additional hour, and then transferred into a 2 ml vial. Immunofluorescence staining with an antibody against brain-specific homeobox/POU domain protein 3A (Brn-3a) was performed similar to the methods previously described [12]. Briefly, retinas were permeated in 0.5% Triton X-100 by freezing for 15 minutes at -70°C, rinsed in fresh Triton X-100, and incubated overnight at 4°C with a primary goat anti-BRN3A (C-20) antibody (Santa Cruz Biotechnology Inc., Santa Cruz, CA) diluted 1:100 in blocking buffer. Retinas were then washed 3 times in PBS and incubated for 2 hours at room temperature with the secondary antibody (Alexa Fluor 594-AffiniPure Rabbit Anti-Goat IgG; Jackson ImmunoResearch Laboratories Inc., West Grove, PA) diluted in blocking buffer. Finally, retinas were thoroughly washed in PBS and mounted vitreal side up on slides and covered with antifading solution. The slides were then imaged on Olympus BX63 fluorescent microscope (Olympus America, Center Valley, PA) with a motorized stage using x40 objective, Olympus DP72 camera and a Texas Red filter cube. Eighth images were acquired for each retina: four images acquired at a distance of 0.9 mm from the optic nerve head, one at each quadrant (inner images) and four were acquired at distance of 1.8 mm from the optic nerve head, one at each quadrant (outer images) (Fig 1). The acquired stacks of images were then exported and subsequently combined into one frame in ImageJ [13]. After background adjustment, the images were converted to 8-bit format and then adjusted for contrast, brightness, and threshold. Larger areas where the cells were perceived as fused were subdivided using a watershed procedure. The RGC nuclei were counted with an automated counting algorithm in ImageJ within the cell nucleus size range of 10 to 300 μm^2^.

#### Estimating cellularity in the optic nerve

Cellularity in the optic nerve tissue was estimated as described before [10]. Briefly, optic nerve cell nuclei were counted using ImageJ software. Images were converted into 8-bit gray-scale images, the background was subtracted, brightness and contrast were manually adjusted and the image was converted into a binary format with a manually optimized threshold. Rectangular areas equivalent in size to 40x microscopic field were generated along the entire length of the optic nerve such that they did not overlap but incorporated as much surface area of the optic nerve as possible. Cells were counted and measured using the particle analyzer included in ImageJ. The average minimum, maximum, perimeter, circularity, percent area, roundness, solidity, and count for each box were recorded. For boxes that encompassed areas outside the optic nerve boundaries, the region of interest including only the optic nerve tissue within the rectangular area was measured and recorded with the data. Cell density (in cells per square millimeter) was calculated for each area based on cell nuclei count and the area of region of interest. The mean value was used for statistical comparison.

#### Immunohistochemistry

Immunohistochemistry of the optic nerve sections were carried out as described before [10]. Briefly, optic nerve specimens were obtained, processed, and mounted on slides, deparaffinized in xylene and rehydrated in a decreasing gradient of ethanol concentration. The sections were then rinsed in water and subsequently incubated at room temperature in a solution of endogenous peroxidase blocking solution for 30 minutes. After rinsing, sections were treated with target retrieval solution and incubated with the protein block solution from the ABC Elite Kit (Vector Laboratories, Burlingame, CA) for a period of 1 hour in a humid chamber at room temperature. Sections were stained in batches with primary antibodies raised against CD45 (CD45, 1:500; AbD Serotec^®^, Raleigh, NC, MCA1388) for receptor-type tyrosine-protein phosphatase C differentially expressed in infiltrating macrophages, and ionized calcium-binding adaptor molecule 1 (Anti-Iba1, 1:5000; Abcam, Cambridge, MA, ab5076) for microglia. As a negative control, one section was incubated with all reagents except the primary antibody. CD45 expression is low in quiescent microglia and increases during microglial activation. Tissue sections stained with CD45 and Iba-1 were subjected to antigen retrieval with modified citrate buffer (Dako, Glostrup, Denmark, S1699). Endogenous peroxidase activity was quenched with a 15 minute H_2_O_2_ treatment (3% in water). Each section was rinsed and incubated with the appropriate blocking buffer for 30 minutes before applying the appropriate primary antibody overnight at 4°C. The diluted biotinylated secondary antibody from the ABC Elite Kit was then applied on each glass slide. Antibodies were detected using the avidin-peroxidase complex, and labeling was revealed after incubating the sections in 3,3-diaminobenzidine (DAB) peroxidase solution for 3 minutes.

### Sample preparation for proteomic and lipidomic analyses

Single optic nerves were thawed, homogenized via bath sonication and sequential freeze-thaw cycles in dry ice in high salt PBS (1M NaCl) supplemented with a protease-inhibitor cocktail tablet (Roche Diagnostics Corporation, Indianapolis, IN). Phospholipid internal standards dissolved in methanol di-14:0 PE (phosphatidylethanolamine), di-14:0 PC (phosphatidylcholine), 17:0 SM (sphingomylein), 14:0 LPE (lysophosphatidylethanolamine), 14:0 LPC (lusophosphatidylcholine), and di-16:0 PI (phosphatidylinositol) (Materya LLC, Pleasant Gap, PA) were added. The homogenates were then clarified by centrifugation for 15 minutes at 20,000 x g RCF at 4°C. Proteins were precipitated in both the resultant supernatant and pellet by addition of 7 volumes of chilled mass-spectrometry grade methanol (Sigma-Aldrich, St Louis, MO). The methanolic supernatants were combined in a new tube, and the pellets were then extracted with 200 μl of 2:1 chloroform:MeOH to extract lipids. The MeOH and chloroform-MeOH extracts were combined and used for lipid analysis, and the pellets were used for proteomic analyses.

### Proteomic analysis

The precipitated proteins were resuspended in 5 μl of LC-MS reduction/alkylation/digestion buffer (1 mM tris(2-carboxyethyl)phosphine [TCEP], 2 mM chloroacetamide, 2.5% w/v sodium deoxycholate (SDC), and 20 mM triethylammonium bicarbonate (TEAB), pH 8.0 (Sigma Aldrich), and incubated at 37°C for 30 minutes. The samples were then diluted to 25 μl with 20 mM TEAB, pH = 8.0 and 200 ng of sequencing grade trypsin (Promega, Madison, WI). Digestion was performed overnight at 37°C. The resultant tryptic peptides for each sample were derivatized with Tandem Mass Tags (TMT Mass Tagging Kit, Thermo Fisher Scientific, Waltham, MA) according to the manufacturer’s instructions. Following labeling, the samples were pooled according to desired sample comparisons (r-mTBI/r-sham) for subsequent identification and quantification, and taken to dryness. The samples were resuspended in 50 μl of 1% formic acid, 99% water to remove SDC from pooled peptide groups. The samples were clarified by centrifugation, and the SDS-reduced supernatant was transferred to a new tube. 200 μl of MS grade ethyl acetate (Fisher Scientific) was used to extract the remaining SDC from the samples, which were again taken to dryness following removal of the ethyl acetate (SDC-containing) upper phase. The peptides were de-salted using C18 ZipTip pipette tips (EMD Millipore, Billerica, MA) according to the manufacturer’s instructions, taken to dryness using a vacuum centrifuge, and resuspended in 20 μl of 0.1% v/v formic acid in water.

An Easy UPLC 1000 (Thermo Scientific) interfaced with a Q-Exactive Orbitrap mass spectrometer (Thermo Scientific) under the control of Xcalibur 2.4 software (Thermo Scientific) was used to generate proteomic data. Approximately 500 ng of tryptic peptides from each pooled sample group was loaded onto a 75 μm x 50 cm C18 reversed-phase (2 μm particle) Acclaim RSLC PepMap column (Thermo Scientific) and separated over a 4.5 hour linear gradient of increasing acetonitrile, from 2–40% at 250 ng/min. The column temperature was held at 40°C using a PST-BPH20 Butterfly column heater (Phoenix S&T, Chester, PA). Data-dependent acquisition (DDA) settings for the Q-Exactive instrument settings were as follows: Full scan MS resolution = 150,000 FWHM at 200 m/z, full scan range = 380–1,250 m/z, isolation width = 1.2 m/z, HCD collision energy (rCE) = 29, a minimum m/z setting of 100 m/z was used for all MS^2^ spectra, MS^2^ resolution = 35,000, dynamic exclusion = 180 seconds, and a Top15 (High/low) duty cycle was used for precursor ion selection. These settings, chiefly the narrow isolation window and the ultra-long gradient, were used to minimize the deleterious effects on quantitative accuracy that result from co-isolation of isobaric precursors during fragmentation without resorting to MS^3^-based methods.

### Data Processing and Statistical Analysis of proteomics data

Samples were analyzed by a Q Exactive benchtop LC-MS/MS (Thermo Scientific). PMi Preview software (PMi Software, Dublin, Ireland) was used to survey the data files and if necessary add other modifications to the search criteria. Also, Preview results were used to choose the precursor and fragment ion mass tolerances (4 ppm, 0.02 Da, respectively), as well as dynamic modifications. The following settings were used to search the data using SEQUEST and BYONIC as the search algorithm; dynamic modifications; Oxidation / +15.995 Da (M), Methyl / +14.016 Da (E), Deamidated / +0.984 Da (N, Q), static modifications of TMT 6plex / +229.163 Da (N-Terminus, K), Carbamidomethyl +57.021 (C). The False discovery rate (FDR) was set to 0.01 in both search engines and peptides passing this cutoff value were exported to JMP 8.0.2 software (SAS Institute, Cary, NC) for data cleaning and statistical analysis. Only unique peptides were considered for quantification purposes. Proteins identified with 2 or more peptides were used for the quantitative analysis. TBI/sham ratios were calculated after *ln* transformation of the raw ion counts. The ratios were normalized by central tendency normalization where medians were used. After the median of a peptide from multiple fractions was calculated, one sample t-test was used to test if the sample mean was equal to zero. The multiple-testing correction as per [14] was applied to identify a “top tier” of significant proteins and prevent identification of false positives at a false discovery rate of 5 percent.

### IPA analysis

Data were uploaded and analyzed through the use of QIAGEN’s Ingenuity^®^ Pathway Analysis (IPA^®^, QIAGEN Inc., Redwood City, CA) software to examine known molecular pathways affected by the injury (default settings were used unless stated otherwise). Briefly, the Ingenuity knowledgebase comprises a repository of proteins/genes that are grouped based on reported biological interactions and functional relationships. Once a dataset is uploaded to IPA (e.g. in this case, proteins extracted from optic nerve tissue which demonstrated significant TBI-dependent changes in expression compared to sham controls), the proteins are mapped onto biological functions and pathways in IPA from which the biological relevance of the response can be inferred. The assignment of each uploaded protein to particular biofunctions and pathways is determined by IPA using published literature and scientific databases. IPA Downstream Effects Analysis was used to identify functions that are expected to increase or decrease, depending on the observed protein regulation changes in the dataset. Downstream Effects Analysis is based on expected causal effects between proteins and functions; the expected causal effects are derived from the literature compiled in the Ingenuity^®^ Knowledge Base. The analysis examines proteins in the dataset that are known to affect functions, compares the proteins' *direction of change* to expectations derived from the literature, then issues a prediction for each function based on the direction of change. The direction of change is the protein expression in the experimental samples relative to a control.

Z-score is used to establish if the direction of the regulation is statistically significant.

Canonical pathways (biological pathways covered by IPA using established scientific databases/literature) most significant to the dataset are determined based on: 1) The ratio of the number of proteins mapping to a pathway divided by the total number of proteins represented in that particular pathway, and 2) a right-sided Fisher’s exact test is applied to calculate a p-value (p < 0.05). The “Core Analysis” function was used to generate biological networks. The cut-off score for considering network inclusion in the analysis was set to 10.

### Lipidomic analysis

The lipid extracts containing added phospholipid internal standards described above (see Materials and Methods –Proteomic analysis) were dissolved in 50 μl 2-propanol and 10 μl injected for analysis by normal phase LCMS to identify and quantify the molecular species of sulfatides, PI, PE, PC, SM, LPC and LPE. As described previously [15], lipid separation was achieved by normal phase high-pressure liquid chromatography using a 1 mm ID X 10 cm column packed with Pinnacle II 3 μm silica particles (Restek, Bellefonte, PA). A solvent gradient was run from 10% solvent B (80% methanol, 10 mM formic acid, 5 mM ammonium hydroxide) in solvent A (chloroform-acetonitrile, 2:1) to 55% B in 12 min with a 5 min hold at the final conditions. The flow rate was 50 μl/min with the column temperature at 40°C. Mass spectrometry was performed using a LTQ-XL linear ion trap mass spectrometer (Thermo-Fisher, Waltham, MA). Alternant positive ion and negative ion spectra were acquired from m/z 75 to 1200 with in-source collision induced dissociation (SCID) relative energy of 25. All spectra were acquired with a 200 ms maximum ion time and by summing 5 microscans. Mass spectra were summed over the chromatographic peak for each PL class, converted to a spectral listing with a threshold of 0.01% base ion intensity, exported to a Microsoft Excel spreadsheet (Microsoft, Redmond, WA) and then the molecular species identified and concentrations calculated using the LipidomeDB online data calculation utility (University of Kansas, Lawrence, KS; http://129.237.137.125:8080/Lipidomics/) with reference to the added internal standards [16]. Data-dependent MS/MS analyses were also performed under the same LCMS conditions to obtain fatty acid and fatty ether composition of the major lipid molecular species detected. Because sulfatide internal standard was not added to the samples, sulfatide molecular species concentrations were calculated using extracted chromatogram peak area ratios compared to that of the PI internal standard (without correction for response differences) and are thus relative and only used for comparison of levels between the experimental groups.

### Statistical analyses

Comparison between groups was done using Student t-test with Welch’s correction. A value of 0.05 was used as a significance level to reject the null hypothesis in all tests. GraphPad Prism 6.0 (Graph Pad Software Inc., La Jolla, CA) was used for graphing purposes.

## Results

### Neuropathological analysis

Despite the fact that no fixative was used for perfusion at the time of anesthesia, there was only minimal retinal loss and the retinas were well-preserved for image analysis. Examination of retinal flatmounts demonstrated a relative reduction in the total number of the Brn-3a-positive retinal ganglion cells in mice subjected to TBI in the inner retinal locations (closer to the optic disc) from 228.1 ± 31 cells/field for r-sham to 181.4 ± 71.4 for r-mTBI—a 20.5% decrease (p = 0.019, t-test). These values corresponded to an average cell density of 2,368 ± 322 Brna-3a-positive RGC cells/mm^2^ for r-sham, a value which is close to the one found by Galindo-Romero et al.[12] using naïve wild type mice and the same method of staining and accounting for location of measurement (our inner retinal samples correspond to their “medium” samples, while our outer samples correspond to their “periphery” samples). Similarly, there was an average of 31.3% decrease of cell density for the average of the four outer retinal locations (r-sham: 198.3 ± 59.3; r-mTBI: 136.3 ± 59.7; p = 0.006, t-test) (Fig 1).

**Fig 2:**
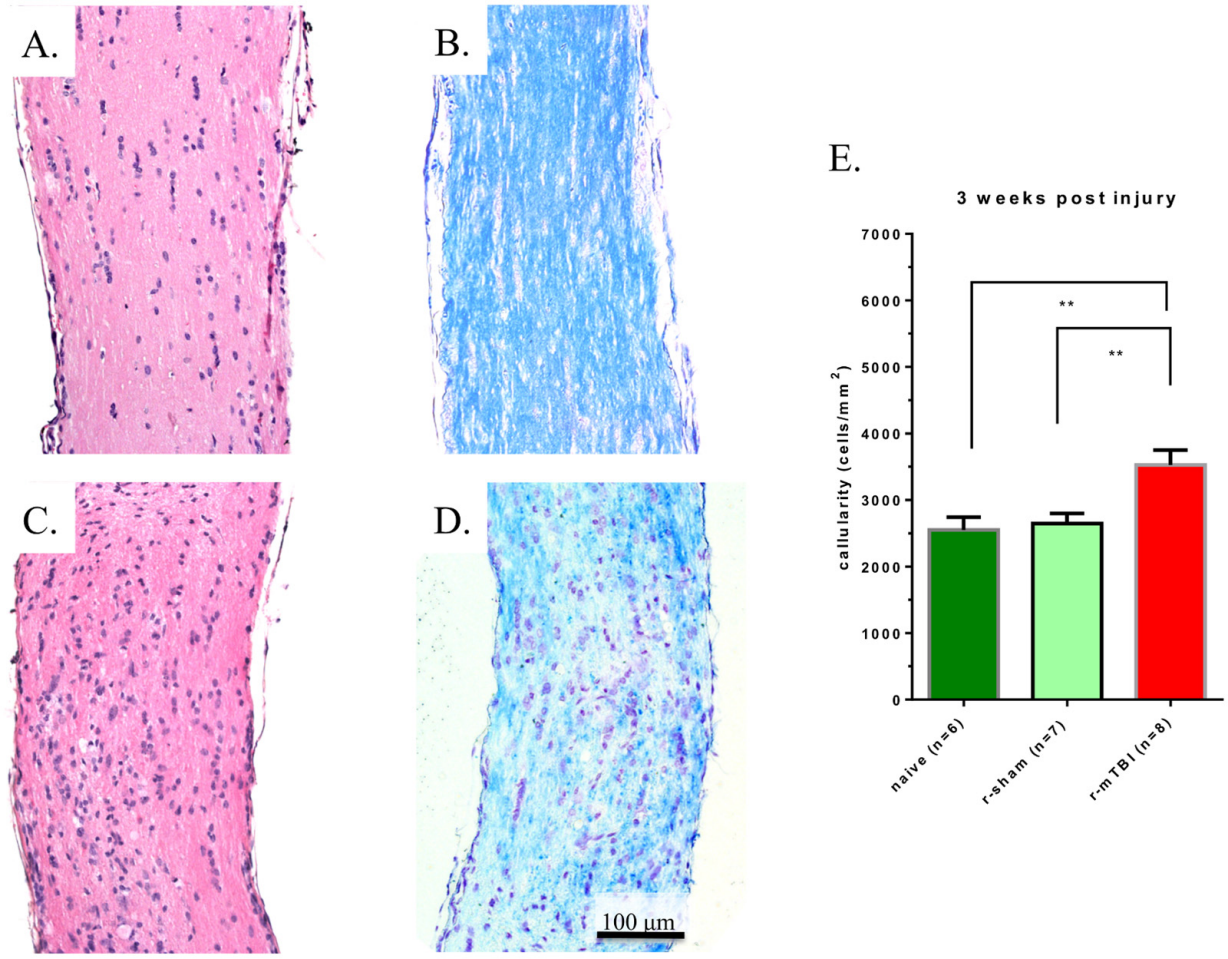
Representative longitudinal cross sections of optic nerves 3 weeks after injury. Optic nerves stained with H&E for repetitive anesthesia alone (r-sham) (A), and r-mTBI (C) mice. There is an overall increase in the number of nuclei in the optic nerve of the (r-mTBI) mouse compared with the r-sham and naïve mice (p<0.01, Mann-Whitney test) (E). Optic nerves stained with Luxol Fast Blue/Cresyl Violet (LFB/CV) from r-sham (B), and r-mTBI (D) mice. Panel D reveals an area of reduced LFB staining indicating focal demyelination.

When comparing H&E stains of longitudinal sections of optic nerves from mice after r-sham vs. mice after r-mTBI, a larger number of nuclei were noted in the latter (Fig 2A and 2C). Systematic counting of the nuclei confirmed the visual impression and demonstrated increased cellularity, from an average cell density of 2,646 ± 404 cells/mm^2^ to an average cell density of 3,526 ± 632 cells/mm^2^, (24.9% increase; p < 0.01, Mann-Whitney test). Of note, there was no difference in cellularity between the r-sham optic nerve samples and the optic nerve samples from naïve mice, which served as an additional control (naïve mouse samples—2,250 ± 469 cells/mm^2^; p > 0.05, Mann-Whitney test) (Fig 2E). Myelin staining with Luxol Fast Blue (LFB) showed focal areas of incipient demyelination, characterized by reduced LFB staining which was more pronounced in the distal third of the optic nerve (Fig 2B and 2D). Although mononuclear infiltrates were present, we did not observe the presence of foamy macrophages or signs “cavernous degeneration” as observed at 10–13 weeks post injury in our previous work [10].

**Fig 3:**
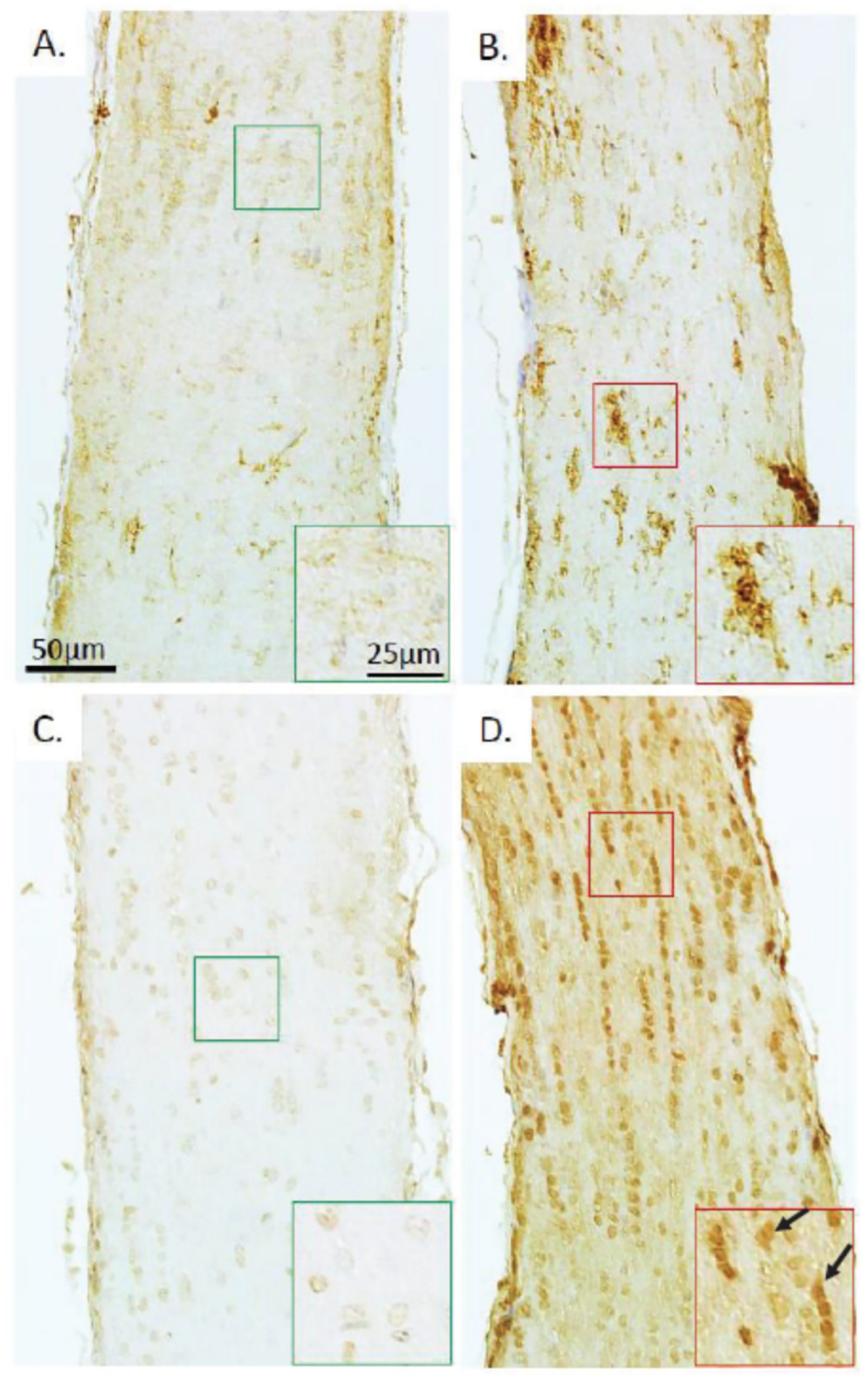
Leucocyte infiltration and microglial activation in optic nerve after r-mTBI. Staining for leucocyte marker CD45 of sections of optic nerves 3 weeks after repetitive anesthesia alone (r-sham) (A) and r-mTBI (B). Ionized calcium-binding adaptor molecule 1 (Iba-1) immunostain of longitudinal sections of optic nerves at the same time point 3 weeks after repetitive anesthesia alone (r-sham) (C) and r-mTBI (D). Higher magnification insets provide further detail, in particular a CD45+ cluster of cells (inset in panel B) and triangularly shaped iba-1+ cells (inset in panel D, arrows).

To investigate the cellular origin of the increased cellularity after r-mTBI, optic nerves were stained for CD45 and Iba-1, markers for leucocytes and microglia, respectively. A slight increase in leukocyte presence was noted, while there was a marked increase in the expression of Iba-1, suggestive of microglial cells as the main driver for increased cellularity (Fig 3). The areas of increased presence of leucocytes and microglia were overlapping with the areas of more pronounced myelin loss in the central part of the optic nerve. Staining with S100B antibodies, which would have indicated astrocytosis and axonal proliferation, did not reveal any increase in staining in r-mTBI optic nerves (data not shown).

### Proteomic analyses

Qualitative and quantitative proteomic analysis of optic nerve preparations identified 540 proteins of which 57 were found to be significantly dysregulated in r-mTBI compared to r-sham, after multiple test correction. Of these, 29 proteins were upregulated and 28 were downregulated (Tables 1 and 2, S1 Table).

**Table 1:** Proteins significantly upregulated in optic nerve tissue 3 weeks after r-mTBI. Proteins were ranked according to the level of upregulation. Location and type according to IPA. Symbols as per UniProt nomenclature.

#	log ratio	% increase	Entrez Gene Name	Symbol	Accession number	In myelin
1	0.082	8.50%	heat shock protein 90kDa beta (Grp94), member 1	HSP90B1	P08113	
2	0.091	9.50%	vimentin	VIM	P20152	☑
3	0.106	11.20%	peroxiredoxin 1	PRDX1	P35700	☑
4	0.127	13.50%	eukaryotic translation elongation factor 1 alpha 1	EEF1A1	P10126	☑
5	0.133	14.20%	ribosomal protein S6	RPS6	P62754	☑
6	0.154	16.60%	AHNAK nucleoprotein	AHNAK	E9Q616	
7	0.155	16.80%	annexin A2	ANXA2	P07356	☑
8	0.167	18.20%	filamin A, alpha	FLNA	B7FAV1	
9	0.17	18.50%	asparaginase like 1	ASRGL1	Q8C0M9	☑
10	0.187	20.60%	leucine aminopeptidase 3	LAP3	Q9CPY7-2	☑
11	0.194	21.40%	actin, beta	ACTB	P60710	☑
12	0.208	23.10%	complement component 4B (Chido blood group)	C4A/C4B	P01029	
13	0.216	24.10%	ribosomal protein S3	RPS3	P62908	☑
14	0.234	26.40%	adenosylhomocysteinase-like 1	AHCYL1	Q80SW1	
15	0.234	26.40%	peptidylprolyl isomerase B (cyclophilin B)	PPIB	P24369	☑
16	0.254	28.90%	filamin C, gamma	FLNC	Q8VHX6-2	
17	0.257	29.30%	quinoid dihydropteridine reductase	QDPR	Q8BVI4	☑
18	0.276	31.80%	glutamate-ammonia ligase	GLUL	P15105	☑
19	0.276	31.80%	moesin	MSN	P26041	☑
20	0.287	33.20%	heat shock 27kDa protein 1	HSPB1	D3YZ06	
21	0.293	34.00%	histone cluster 1, H2bk	HIST1H2BK	Q8CGP1	
22	0.324	38.30%	glucosamine-6-phosphate deaminase 1	GNPDA1	O88958	
23	0.342	40.80%	apolipoprotein E	APOE	P08226	☑
24	0.372	45.10%	*Protein inferred from homology* †		A8DUK4	
25	0.378	45.90%	albumin	ALB	P07724	☑
26	0.393	48.10%	*predicted gene*, *17669; Protein predicted*	Gm17669	F6QL70	
27	0.401	49.30%	calponin 3, acidic	CNN3	Q9DAW9	
28	0.45	56.80%	myristoylated alanine rich PKC substrate	Markcs	P26645	☑
29	0.616	85.20%	cathepsin D	CTSD	P18242	☑

☑—protein detected in mouse myelin [17];

^†^—protein not identified in the IPA database; according to UniProt (The UniProt Consortium; http://www.uniprot.org/), it represents a beta-globin, an iron-binding protein typically a part of the hemoglobin molecule.

**Table 2:** Proteins significantly downregulated in optic nerve tissue 3 weeks after r-mTBI. Proteins were ranked according to the level of downregulation. Location and type according to IPA. Symbols as per UniProt nomenclature.

#	log ratio	% decrease	Entrez Gene Name	Symbol	Accession number	in myelin
1	-0.096	-9.2%	spectrin, alpha, non-erythrocytic 1	SPTAN1	P16546	☑
2	-0.107	-10.1%	dihydropyrimidinase-like 2	DPYSL2	O08553	☑
3	-0.109	-10.3%	spectrin, beta, non-erythrocytic 1	SPTBN1	Q62261	☑
4	-0.14	-13.1%	spectrin, alpha, non-erythrocytic 1	SPTAN1	A3KGU5	
5	-0.149	-13.8%	myelin basic protein	MBP	P04370-9	☑
6	-0.191	-17.4%	tubulin, beta 2A class IIa	TUBB2A	Q7TMM9	☑
7	-0.197	-17.9%	heat shock 70kDa protein 12A	HSPA12A	Q8K0U4	☑
8	-0.21	-18.9%	collagen, type VI, alpha 3	COL6A3	E9PWQ3	
9	-0.223	-20.0%	collapsin response mediator protein 1	CRMP1	P97427	☑
10	-0.225	-20.1%	microtubule-associated protein 1A	MAP1A	A2ARP8	
11	-0.228	-20.4%	collagen, type VI, alpha 1	COL6A1	Q04857	
12	-0.237	-21.1%	erythrocyte membrane protein band 4.1-like 3	EPB41L3	Q9WV92-7	☑
13	-0.251	-22.2%	collagen, type VI, alpha 3	COL6A3	J3QQ16	
14	-0.255	-22.5%	myelin associated glycoprotein	MAG	P20917-2	☑
15	-0.259	-22.8%	ATPase, Na+/K+ transporting, alpha 3 polypeptide	ATP1A3	Q6PIC6	☑
16	-0.26	-22.9%	dihydropyrimidinase-like 5	DPYSL5	Q9EQF6	☑
17	-0.268	-23.5%	2',3'-cyclic nucleotide 3' phosphodiesterase	CNP	P16330	☑
18	-0.275	-24.0%	peripherin	PRPH	P15331-3	
19	-0.305	-26.3%	calbindin 2	CALB2	Q08331	☑
20	-0.33	-28.1%	neurofilament, heavy polypeptide	NEFH	P19246	☑
21	-0.399	-32.9%	internexin neuronal intermediate filament protein	INA	P46660	☑
22	-0.4	-33.0%	A kinase (PRKA) anchor protein 12	AKAP12	Q9WTQ5-2	☑
23	-0.417	-34.1%	synuclein, gamma	SNCG	Q9Z0F7	
24	-0.426	-34.7%	neurofilament, medium polypeptide	Nefm	P08553	☑
25	-0.436	-35.3%	microtubule-associated protein 1B	MAP1B	P14873	☑
26	-0.496	-39.1%	tubulin, alpha 4a	TUBA4A	P68368	☑
27	-0.506	-39.7%	neurofilament, light polypeptide	NEFL	P08551	☑
28	-0.626	-46.5%	AHNAK nucleoprotein 2	AHNAK2	E9PYB0	

☑—protein detected in mouse myelin [17]

Data for all of the significantly regulated proteins were uploaded to IPA, which identified and mapped 56 of them, while one protein (A8DUK4) was missing from the IPA database. Core Analysis revealed that 36 canonical pathways were predicted to have responded significantly to r-mTBI (S2 Table).

Further analysis with IPA Downstream Effects indicated that three function annotations were significantly changed:

1. *Depolymerization of microtubules* (function that belongs to the category Cellular Assembly and Organization, Cellular Compromise, Cellular Function and Maintenance) was upregulated (activation z-score 2.20; p <0.0001) with all five proteins involved (APOE, CRMP1, DPYSL2, MAP1A, MAP1B) having an increased expression, consistent with increased depolymerization of microtubules
2. *Disassembly of filaments* (function that belongs to the category Cellular Compromise) was upregulated (z-score 2.41; p <0.0001) with all six proteins (APOE, CRMP1, DPYSL2, MAP1A, MAP1B, VIM) having expression direction (either increased or decreased) consistent with increased disassembly of filaments
3. *Quantity of neurons* (function that belongs to the category Nervous System Development and Function, Tissue Morphology) was downregulated (z-score 2.20; p = 0.0003) with five out of six proteins (CTSD, FLNA, NEFH, NEFL, NEFM, SNCG) having expression direction (either increased or decreased) consistent with decreases in quantity of neurons

In addition, IPA analysis generated four molecular networks including affected proteins with a cut-off score of 10 or more (see also IPA Analysis in Materials and Methods):

1. *Cellular Assembly and Organization*, *Cellular Compromise*, *Cellular Function and Maintenance*; score = 46 (S1 Fig)
2. *Cancer*, *Lipid Metabolism*, *Molecular Transport*; score = 32 (S2 Fig)
3. *Hereditary Disorder*, *Skeletal and Muscular Disorders*, *Cell Morphology*; score = 24 (S3 Fig)
4. *Behavior*, *Cell Morphology*, *Cellular Function and Maintenance*; score = 10 (S4 Fig).

### Lipidomic analyses

**Fig 4:**
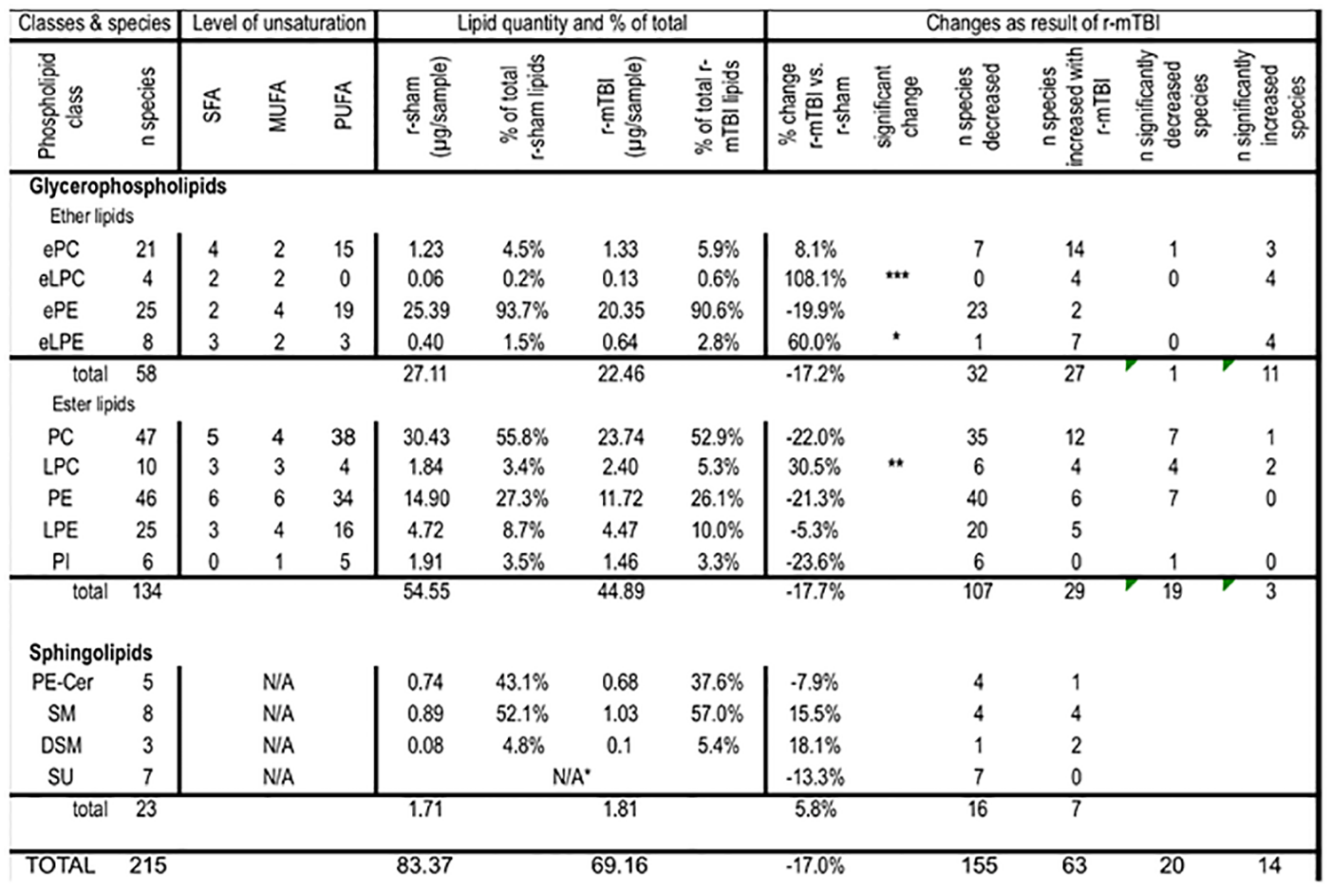
Summary of the lipid classes and species detected in optic nerve tissue in the current study.

Overall, 213 lipid species were identified to be present in the optic nerves above the lower limit of quantitation, distributed among 13 different glycerophospholipid and sphingolipid classes. The glycerophospholipids included ether lipid classes: ether phosphatidylcholine (ePC), ether lysophosphatidylcholine (eLPC), and ether phosphatidylethanolamine (ePE), and ester lipid classes: phosphatidylcholine (PC), lysophosphatidylcholine (LPC), phosphatidylethanolamine (PE), lysophosphatidylethanolamine (LPE), and phosphatidylinositol (PI). Sphingolipids included the following classes: ceramide phosphoethanolamine (PE-Cer), sphingomyelin (SM), dihydrosphingomyelin (DSM), and sulfatide (SU) (Fig 4, S3 and S4 Tables). The total amount of phospholipids in the optic nerve tissue was decreased by 17.6% after r-mTBI, but this difference was not statistically significant. Out of the 13 classes, two ether lysophospholipid classes showed significant increase in *total* lipid quantity after r-mTBI (eLPC, eLPE) and in some individual molecular species. Furthermore, in five other classes (ePC, PC, PE, PLE, PI), *individual* lipid species showed either a significant increase or decrease in lipid quantity (Fig 4). The total amount of ether phospholipids and ester phospholipids were decreased to a similar extent (-17.2% vs. -18.6%, respectively) after r-mTBI, although for neither of the classes the decrease was statistically significant. However, in terms of change in individual species the pattern of change within the two classes was very different. Thus, 11 out of the 12 individual species (91.7%) significantly dysregulated after TBI showed increase in quantity after TBI for ether lipids compared to two out of the 22 species (9.1%) for ester lipids (Fig 4).

In contrast to the glycerophospholipids, sphingolipids did not show any statistically significant change, neither by overall amount nor in terms of change in individual species (Fig 4). Changes in some of the individual lipid classes are presented below.

### Ether phospholipids

**Fig 5:**
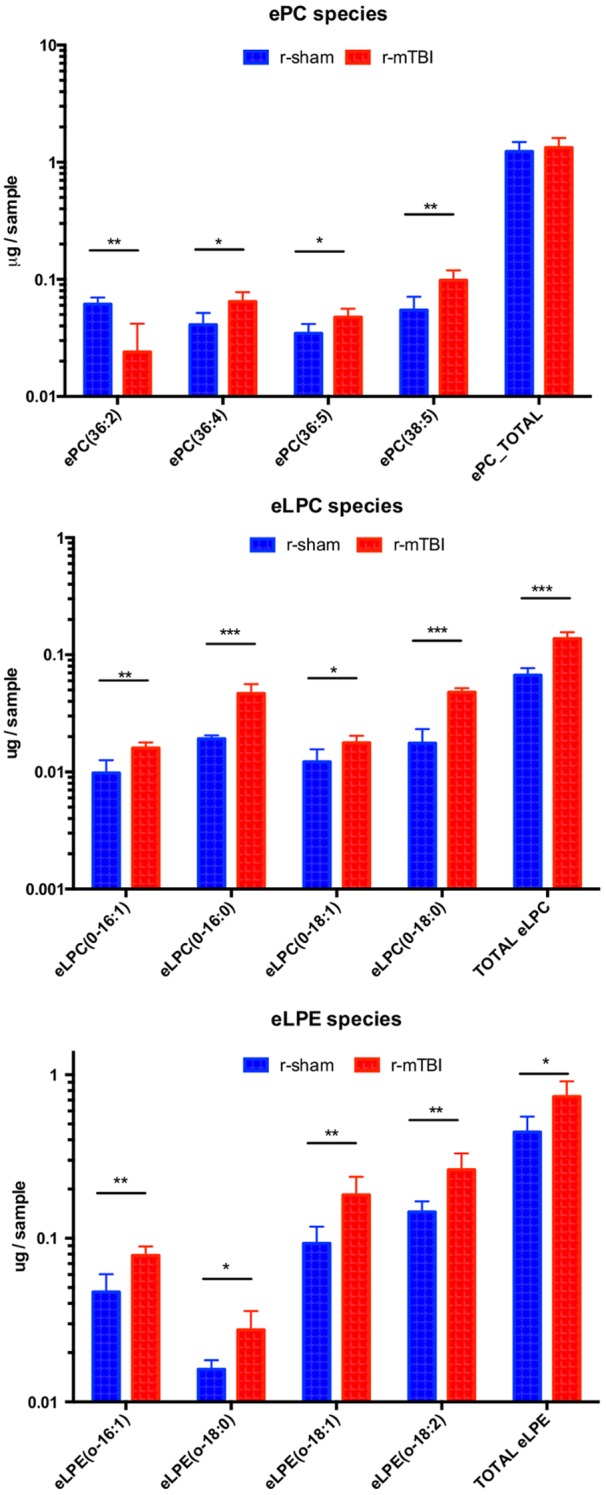
Quantitative assessment of ether phosphatidylcholine (ePC), ether lysophosphatidylcholine (eLPC) and ether lysophosphatidylethanolamine (LPE) species in optic nerve samples at 3 weeks after injury. Significant changes in individual molecular species together with changes in the total amount of lipids in the respective classes are shown. Upper panel—changes in ePC, middle panel—changes in eLPC, lower panel—changes in eLPE species. Blue bars represent total amount of phospholipids (μg/sample) in mice after r-sham, red bars—after r-mTBI. Asterisks indicate statistical significance between groups: *—p < 0.05; **—p <,0.01; ***—p < 0.001.

*Ether phosphatidylcholines (ePC)*. Despite the fact that the overall amount of ePC was increased by only 8.1% and this change was not statistically significant (p>0.05, t-test), the majority of the ePC species (14 out of 21, or 66.7%) were increased (Fig 4), and for three species: ePC(36:4), ePC(36:5), and ePC(38:5), this increase was statistically significant, while one species—ePC(36:2) was significantly decreased (Fig 5, S4 Table, Fig 7).

*Ether lysophosphatidylcholines (eLPC)*. Although this class of ether phospholipids was the least abundant of all ether phospholipids (0.6%), it was the most significantly upregulated in quantity of any phospholipid class in this study at 108.1% (p<0.001, t-test), and all four individual species that passed the detection criteria were significantly increased after TBI (Fig 5, S4 Table, Fig 8).

**Fig 6:**
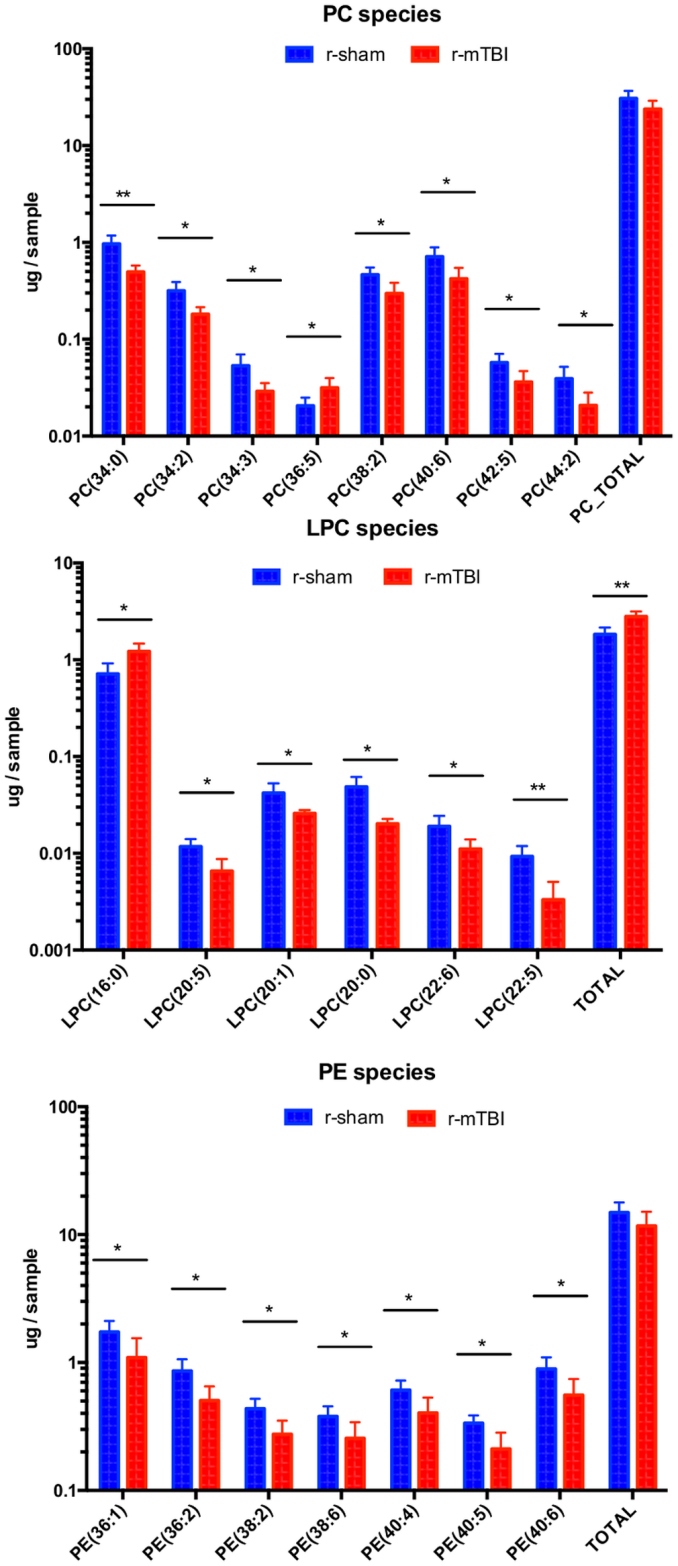
Quantitative assessment of phosphatidylcholine (PC), lysophosphatidylcholine (LPC) and lysophosphatidylethganolamine (LPE) in optic nerve samples at 3 weeks after injury. Significant changes in individual molecular species together with changes in the total amount of lipids in the respective classes are shown. Upper panel—changes in PC, middle panel—changes in LPC, lower panel—changes in LPE species. Color coding and asterisk association same as in Fig 5.

*Ether lysophosphatidylethanolamines (eLPE)*. Similar to the changes observed in eLPC, the overall amount of eLPE, which is another minor ether lipid class (2.8% of all ether phospholipids), was significantly increased after TBI by 60.0% (p<0.05). Of the seven species showing an increase after TBI, in four species the increase was found to be statistically significant: eLPE(0–16:1), eLPE(0–18:0), eLPE(0–18:1), and eLPE(0–18:2) (Fig 6, S4 Table, Fig 8).

### Diester phospholipids

*Phosphatidylcholines*. The total amount of PC decreased at 3 weeks after r-mTBI (-22%), although this change was not statistically significant (Fig 4). Out of the 47 PC species that could be detected in optic nerve samples, most were decreased (35 species or 74%) after r-mTBI (Fig 4, S4 Table), and for seven species the decrease was significant: PC(34:0), PC(34:2), PC(34:3), PC(38:2), PC(40:6), PC(42:5), PC(44:2) (Fig 5), while only one species showed a significant increase—PC(36:5) (Fig 8, S4 Table).

*Lysophosphatidylcholines*. The total amount of LPC was significantly increased after r-mTBI (30.5%, p<0.05). It should be noted that this was the only class of ester phospholipids that showed an overall increase in quantity after injury (Fig 4). Additionally, this increase was due to the increase in the most abundant molecular species,—LPC(16:0) (39.1% of all LPC; 70.4% increase after TBI), while all five other species that were significantly dysregulated, LPC(20:5), LPC(20:1), LPC(20:0), LPC(22:6), and LPC(22:6), showed a decrease in quantity (Fig 5, S4 Table, Fig 8).

*Phosphatidylethanolamines*. The total amount of PE was decreased by 21.5% after TBI, although this decrease was not statistically significant. Furthermore, 38 out of the 44 species (86.4%) showed a decrease, although only for seven species (15.2%) the decrease was statistically significant (Fig 6, S4 Table, Fig 8).

#### Effect of injury on the degree of unsaturation of phospholipid classes

**Fig 7:**
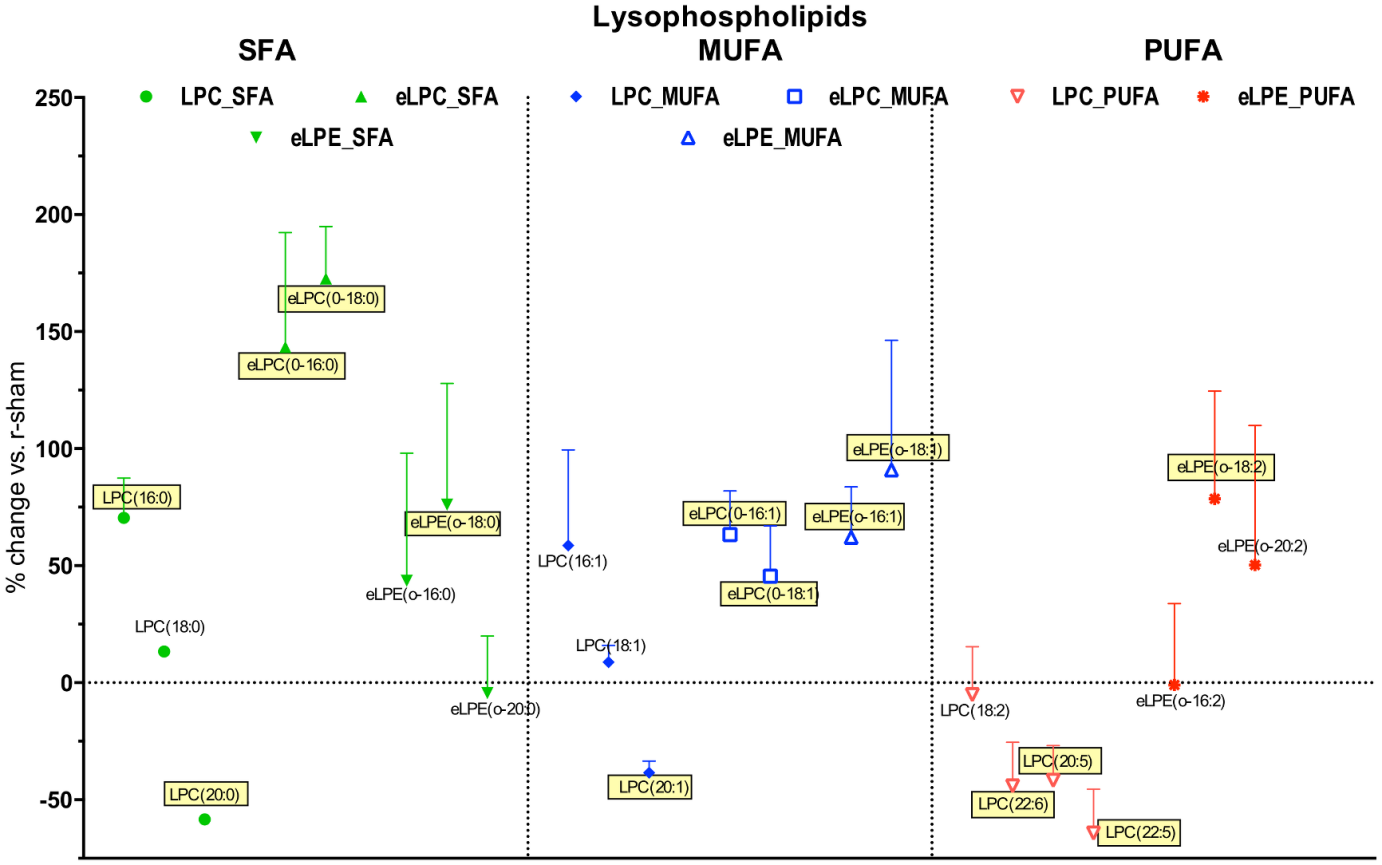
Effect of r-mTBI on lyso-phospholipid species detected in the present study based on degree of unsaturation. Saturated fatty acid species (SFA) are presented in green, monounsaturated fatty acid species (MUFA) are presented in blue, while polyunsaturated fatty acid species (PUFA) are presented in red. Symbols represent mean values of percent change of r-mTBI samples compared to r-sham samples; error bars indicate SEM. Note 1: due to the large number of PUFA species detected, only the ones showing > ±25% change are presented. Note 2: Species names covered with yellow boxes indicate statistical significance (p<0.05).

We then explored the effect of the degree of unsaturation on change in individual phospholipid species after r-mTBI. For the lysophospholipid classes (LPC, eLPC, eLPE) there was a tendency for proportionally more species (more than 50%) to be increased after TBI in the saturated fatty acid (SFA) and monounsaturated fatty acid (MUFA) groups compared to less than half of the species (28.6%) in the polyunsaturated fatty acid (PUFA) group (Fig 6). The opposite tendency was observed in the diacyl-glycerophospholipids (PC, PE, ePC), where there were relatively less species increased in SFA and MUFA groups (35.7% and 27.3%, respectively), while the proportion of increased species remained similar in the MUFA group at 36.8% (Fig 7). This pattern divergence between lysophospholipids and diacyl-glycerophospholipids was even more pronounced when examining only the significantly changed species, with no significantly increased species in SFA and MUFA glycerophospholipids versus four out of five (75%) in the lysophospholipid group.

**Fig 8:**
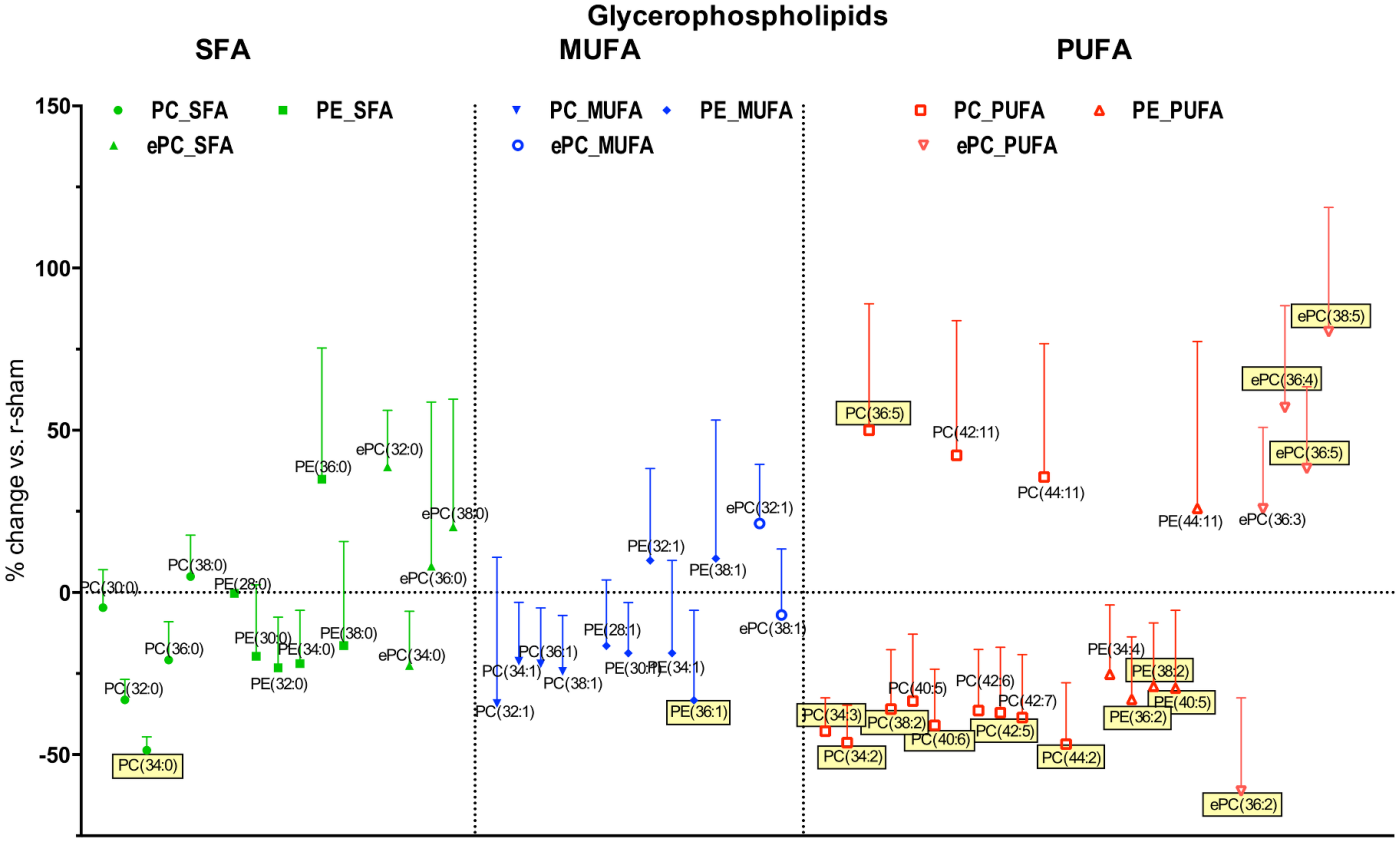
Effect of r-mTBI on diacyl-glycerophospholipid species detected in the present study based on degree of unsaturation. Saturated fatty acid species (SFA) are presented in green, monounsaturated fatty acid species (MUFA) are presented in blue, while polyunsaturated fatty acid species (PUFA) are presented in red. Symbols represent mean values of percent change of r-mTBI samples compared to r-sham samples; error bars indicate SEM. Note 1: due to the large number of PUFA species detected, only the ones showing > ±30% change are presented. Note 2: Species names covered with yellow boxes indicate statistical significance (p<0.05).

## Discussion

### Neuropathological findings

Retinal ganglion cell loss and increased cellularity in the optic nerve were previously described at 10–13 weeks post injury in our closed-head repetitive TBI mouse model [10]. In the current study of the same TBI model at an earlier, 3-week post-injury time point, both phenomena were also present, although to lesser extent. Although we were not able to quantify the precise extent of the RGC loss at the 3-week time point, a rough estimate for it based on local sampling would indicate an ~25% RGC loss. This can be compared with ~67% loss at 13 weeks in the previous study, indicating a progression of the retrograde retinal degeneration over time post injury. Thus, it appears that the rate of RGC loss in our model is slower compared to the RGC loss in some well-established models of *direct* optic nerve injury in mice like intraorbital optic nerve crush injury at 2 weeks (73%) [18], or after a complete optic nerve intraorbital transection (~85%) at 3 weeks [12].

The amount of cellular infiltration in the optic nerve reflected by the level of cellularity in the present study is 24% smaller compared to that reported at 13 weeks (3,526 cells/mm^2^ in the current study vs. 4,361 cells/mm^2^ [10]). Thus, it can be hypothesized that cellular infiltration represents a persistent and likely progressing phenomenon, which could be linked to the ongoing optic nerve degeneration and the resultant RGC loss. As the rate of RGC loss at 3 weeks post injury appears to be considerably smaller compared to 13 weeks post injury, the number of degenerating optic nerve axons is also much smaller. Therefore, activated microglia, whose primary function in optic nerve injury or degeneration is to phagocytose myelin [19], appears to be less activated at 3 weeks vs. 13 weeks post injury. Furthermore, the current study indicates that, at least for the 3-week time point, the majority of the infiltrating cells are likely activated microglial cells as evidenced by the increased Iba-1 staining with very little contribution from leukocytes. Similar microglial responses in the optic nerve were observed in other models of optic nerve injury: in injured mouse optic nerves 7 to 28 days after single injury [20] and 7–35 days after rat optic nerve crush [21].

### Proteomic analysis

#### General impressions

Proteomic analysis of the optic nerve tissue revealed a number of significant changes in protein expression in response to injury. In our IPA Downstream effects analysis, we identified three functions significantly dysregulated at 3 weeks post injury. Two functions were related to cellular compromise and indicated increased “depolymerization of microtubules” and increased “disassembly of filaments”, while the third function was consistent with decrease in “quantity of neurons”.

The fact that the majority of the proteins identified as dysregulated in the current study are identified in mouse myelin (Tables 1 and 2) supports the hypothesis that the main pathological consequences of the repeated head trauma are related to an ongoing process of demyelination and axonal damage. Due to space limitations it is not possible to discuss in detail all dysregulated proteins. Thus, the focus of the discussion to follow will be mostly on changes in the molecules involved in the three significantly dysregulated functions identified by IPA.

*Depolymerization of microtubules* and *Disassembly of filaments* are two major biological functions that are of primary importance in the dynamic maintenance of microtubule integrity. Microtubules are the stiffest structural components of axons and thus may be at risk of damage during TBI, as *in vitro* studies have demonstrated axonal microtubule loss after stretch injury and progressive disassembly of microtubules along the breakage points [22]. Furthermore, axonal cytoskeletal injury appeared early after guinea pig optic nerve stretch injury and led to an increase in myelin dislocations/discontinuities with increasing time post injury (up to 4 weeks) [23].

*Quantity of neurons* is an IPA function that indicates changes in neuronal quantity that most often manifests as cell death. The fact that this pathway was downregulated corresponds to our observed decrease in RGC numbers from the neuropathological analysis. Furthermore, it has been reported that 3 weeks post injury is a time point associated with a delayed wave of increased cell death after optic nerve crush in rats [24], consistent with the findings from the present study.

### Specific findings in proteins related to axonal damage

*Findings related to axonal damage and degeneration*: *neurofilament triplet proteins*, *spectrin*, *tubulin*, *tubulin-associated and microtubule-associated proteins*. Neurofilament light polypeptide (NEFL), neurofilament medium polypeptide (NEFM), and neurofilament heavy polypeptide (NEHP) all belong to the group of neurofilament triplet proteins, major cytoskeletal components of neurons in the CNS, providing mechanical strength and stability to the axons (where they are particularly abundant) and involved in transport or localization of intra-axonal components. Some authors classify peripherin (PRPH) and alpha internexin (INA) also as belonging to the neurofilament family as neuronal-specific intermediate filament proteins [25–27]. Optic nerve transection results in 70–80% decrease in NEFL levels in rat retina at 21 days post injury [28] and the levels of all three neurofilament triplet proteins decrease up to 60 days after optic nerve crush in rats [29]. Similarly, optic nerve levels of both NEFL and NEFH are decreased 0.5 hours to 3 days after optic nerve stretch in mice [30]. Thus, our finding of decreased levels of NEFL, NEFM, NEHP and also PRPH and INA proteins is in line with several works showing changes in neurofilaments in optic nerve and retina after optic nerve injury; for a detailed review see Agudo-Barrioso et al. 2011 [31].

Spectrins are cytoskeletal proteins which are expressed in most organs and tissues. Of particular interest is their role in the axonal cytoskeleton, which was recently revealed to be organized in a ladder-like structure with alternating rings of actin linked together by intervening complexes of spectrin [32]. Spectrin breakdown was observed acutely, and, as a second wave, at four days after stretch injury of the optic nerve in mice [33], but not at 2 weeks post injury. Similarly, spectrin was decreased in the sera at 3 weeks post ischemia-reperfusion retinal injury in rats, in parallel with signs of optic nerve degeneration [34]. We also found a mild decrease in alpha and beta spectrins (SPTAN1, SPTBN1), indicating a continuing breakdown of the optic nerve axonal cytoskeletons. Our finding of parallel spectrin and tubulin decrease is also consistent with similar findings acutely and up to 72 hours post optic nerve stretch injury in mice [30], supporting the involvement of similar mechanisms, but at a later time point post injury.

Alpha- and beta tubulins are members of the globulin protein superfamily and polymerize to form microtubules, a major component of the cytoskeleton, especially in neurons [35]. One representative of each type, tubulin beta 2A (TUBB2A) and tubulin alpha 4A (TUBA4A) were found to be decreased post injury in our model. It is worth noting that neurofilaments bind to tubulin and modulate its polymerization [36], supporting the notion that the decline in neurofilaments and tubulin in our model are interrelated. This is supported additionally by our finding of decrease in tubulin-associated and microtubule-associated proteins. Collapsin response mediator protein 1 (CRMP1) and dihydropyrimidinase-like 2 (DPYSL2, also known as CRMP2) are both tubulin-associated proteins that regulate axonal growth and stabilize microtubules [37, 38]. Limiting the axonal phosphorylation of CRMP2 can preserve the axons of the optic nerve, and thus prevent neuroinflammatory-mediated axonal degeneration [39]. It is possible that the observed decrease in the levels of both proteins in the present study indicate a suppression of the axonal growth signaling. Another interpretation could be that their decrease is the result of tubulin breakdown and phagocytosis. Microtubule-associated proteins 1A (MAP1A) and 1B (MAP1B) both belong to a group of microtubule-associated proteins involved in repeated cycles of tubulin assembly and disassembly and mediate the physical interactions between microtubules and components of the cytoskeleton. Although they appear to share little structural similarity [40], they associate with similar low molecular weight polypeptides [41]. It has been demonstrated that MAP1A is transported along the length of the axon independently of tubulin and contributes to a stationary axonal cytoskeleton [42]. As for MAP1B, its levels can be elevated up to two weeks after optic nerve crush [43]. Our finding that the levels of both proteins are downregulated supports the other observations of decrease in the level of tubulin and tubulin-associated proteins likely reflecting the process of continuous axonal breakdown and degeneration.

*Filamin-A increase*. Filamin-A is a widely expressed protein that regulates reorganization of the actin cytoskeleton [44]. To the best of our knowledge, there have been no prior reports of changes in filamin-A levels after optic nerve or other types of axonal injury. A recent study found that filamin-A plays critical roles in HDAC5-dependent tubulin deacetylation [45], corresponding to the observed decrease in the level of tubulins in our model and indicating a complex mechanism of axonal injury that triggers and sustains the process of optic nerve degeneration post r-mTBI.

*Gamma synuclein decrease*. Gamma synuclein is a protein of unknown function that is expressed exclusively in the retinal ganglion cell axons of the optic nerve [46]. Its levels are decreased in optic nerves of the rat elevated IOP model [47] and in the optic nerve of adult DBA/2J mice, a model of glaucoma associated with elevated IOP [48]. Our results are consistent with these studies and indicate molecular level similarities between optic nerve degeneration after IOP elevation and after mTBI, likely due to RGC death.

*Increase in Cathepsin D*. Cathepsin D is a lysosomal aspartyl protease, active in intracellular protein breakdown, including all three classes of neurofilaments [49], and is present in the optic nerve [50]. Levels of cathepsin D were increased 7 days after transection of the sciatic nerve [51] and 5 weeks after intraocular pressure elevation leading to optic nerve degeneration [52]. In the present study, the significant elevation of cathepsin D levels at 3 weeks post injury is suggestive of continuous degradation of neurofilaments and other axonal components at this time point.

*APOE increase*. Apolipoprotein E is a lipid-binding glycoprotein involved in phospholipid and cholesterol transport and metabolism and is normally expressed in astrocytes and oligodendrocytes in the optic nerve. It has been demonstrated that its levels can be elevated following crush or transection of the optic nerve and may participate in the redistribution of myelin lipids [53, 54]. Our findings are consistent with these observations.

*Vimentin upregulation*. Vimentin (VIM) is a type III intermediate filament protein and an astroglial marker. It has been demonstrated that vimentin is upregulated at 1 and 3 days post optic nerve crush in mice [55] and antibody reactivity against vimentin were increased in rat sera 2–4 weeks after ocular ischemia as a result of elevated intraocular pressure (IOP) in rats [34]. Furthermore, it was shown that in the rat optic chiasma vimentin colocalises with the heat shock protein 27 (HSPB1) and that HSPB1 is significantly upregulated after optic nerve crush up to 7 days post injury [56]. Our observations for optic nerve tissue expression of both VIM and HSPB1 confirm and expand these findings within the scope of our model, supporting an ongoing astroglial activation in the optic nerve at 3 weeks post injury.

#### Other protein changes

Of particular interest may be the observed decrease in myelin basic protein (MBP), myelin-associated glycoprotein (MAG) and 2',3'-cyclic nucleotide 3' phosphodiesterase (CNP or CNPase) in the present study (Table 2). MBP is a major constituent of CNS myelin and mature oligodendrocytes. Whereas the slight decrease in MBP levels could be a direct reflection of an ongoing demyelination [57], the substantial decrease in MAG levels may indicate a reparative process, as an inhibition of the CNS myelin growth is believed to be the main function of this protein [58, 59]. Of note, both proteins appeared to be downregulated to a similar extent (-21% MAG and -18% MBP) in an experimental system with Schwann cells responding to two hours of sustained shear stress [60], suggesting that shear stress may be part of the pathological mechanism in the present model. CNPase is a myelin-associated enzyme expressed exclusively by oligodendrocytes in the CNS [61] and as such would be expected to be decreased in cases of ongoing optic nerve demyelination in parallel to both MBP and MAG [62], an expectation completely confirmed by our observations.

In summary, the proteomic data of optic nerve samples from the present study are suggestive of an ongoing degenerative process in the mouse optic nerve at 3 weeks after r-mTBI with signs of axonal damage and related neuronal (RGC) cell death.

### Lipidomic analysis

Overall, a tendency for decrease in lipid quantity after TBI was observed in the optic nerve samples in the present study. However, the lipid classes that showed a statistically significant change in lipid quantity after injury all demonstrated an increase and were all lysophospholipids (eLPC, LPC, eLPE).

### Changes in phospholipid levels

*LPC and eLPC elevation*. Ether containing lysophosphatidylcholine and ester lysophosphatidylcholine are minor phospholipids in the cell membrane and in the blood plasma with short *in vivo* half-lives. In general, lysophospholipids interfere with lipid membrane structures and ion channel activities and their increased local concentration can be detrimental to neurons and their axons [63]. Numerous studies in the past have shown that application of LPC can cause axon-sparing, segmental demyelination in the peripheral and central nervous system, including the optic nerve [64–66], which can be followed by remyelination [67]. However, none of these studies specified which of the numerous LPC species (or what type of combination of LPC species) was used to induce this process. In our sample set, two LPC species stand out as increased at 3 weeks post injury. The most abundant LPC species, 1-palmitoyl LPC(16:0), has been studied extensively and exhibits potent pro-inflammatory properties, causing eosinophil adhesion [68], monocyte migration [69], elevation in the serum of atherosclerotic patients and in atherosclerotic tissue [70, 71], apoptosis in vascular smooth muscle or endothelial cells [72, 73], and is elevated early (up to 180 minutes) after spinal cord injury in a mouse model [74]. Our finding is the first observation of an increase of LPC(16:0) in CNS tissue at a sub-chronic time point after TBI and, together with our neuropathological data, supports the pro-inflammatory role of this species. The biological activity of this compound may be more complex, as it has been demonstrated that it can prevent neuronal death in an *in vivo* model of transient global ischemia and in an *in vitro* model of excitotoxicity [75], and prevent apoptotic cell death in cultured cerebellar granule neurons [76]. It has been also demonstrated that total LPC is elevated in the cerebrospinal fluid (CSF) of patients surviving moderate to severe TBI [77]. Thus, it would thus be interesting to explore both plasma levels and CSF levels in mice to determine the level of correlation between these values, which may help establish LPC levels as a biomarker for TBI in clinical settings. Another interesting observation in the present study was the significant relative elevation of total eLPC (about three times more pronounced that the elevation of LPC) and the particular increase of the two most abundant eLPC species—eLPC(16:0) and eLPC(18:0). Changes in these species after axonal injury has not been reported before so further *in vivo* experiments are warranted to determine their pro-inflammatory, demyelinating and neurodegenerative properties. Such properties may exist, as studies have demonstrated that complete inhibition of ether lipid synthesis leads to defects of myelination and other axonal abnormalities in the brain [78].

*eLPE elevation*. Little is known about the eLPE biosynthesis, distribution, turnover rate, and functional role in the optic nerve. Generally, it is believed that in adult rodents, myelin ether phospholipids are relatively inactive and represent structural components of myelin [79]. The role of ether lipids in the optic nerve structure and function remains largely enigmatic, although evidence has emerged that they may play an essential part of the myelin biosynthesis and maintenance in this tissue and in the retina [80]. It has been clearly demonstrated that in mice lacking ether lipids, several defects of the optic nerve morphology and physiology were observed, like decrease in the diameter of the myelinated portion of the nerve, increase in length of the non-myelinated portion of the nerve, increase in paranodal length, etc. [78, 81]. Thus, the present study is the first to quantify eLPE levels in the optic nerve and to report their considerable increase (60%) at 3 weeks after r-mTBI, and, as with eLPC, further work is warranted to clarify their role in the biochemical and structural changes taking place in the optic nerve at this and other time points after injury.

*PC*, *PE and PI decrease*. Although PC, PE, and PI had different abundance in our samples (56.6%, 27.6% and 3.6%, of the total amount of ester lipids respectively), they appeared to demonstrate the same tendency for a decrease to a similar extent after injury (-22.0%, -21.5% and -23%, respectively). This tendency for a decrease in these phospholipid classes differs from the changes observed after rabbit optic nerve transection, where PC and PE were significantly increased at a similar time point [82]. As all of these three ester lipid classes are major structural components of myelin, and given the localized nature of the lesion, it is not surprising that they show a tendency to be reduced to a similar extent, however, this change did not show statistical significance.

### Relationship between protein and lipid changes after TBI

Some relationships between the observed changes in the protein and lipid quantities after TBI deserve further attention. Thus, the increase in complement factor C4, which likely indicates an induction of the classical complement pathway, can be related to the noted increase in LPC. Such a relationship could be suggested by *in vitro* experiments, which demonstrated that LPC induced myelin degradation, while a C4-deficient serum was ineffective in the same system [83]. This was further supported by the results of another study, which demonstrated that LPC can directly activate C4 in rat plasma [84]. Elevation of C4 levels was detected in CSF of multiple sclerosis patients [85], implicating a relationship with demyelination. CD4 knockout mice showed less damage and better performance compared to wild type mice in a controlled cortical impact model of TBI [86]. Taken together, these observations suggest a link between LPC and complement activation in TBI, which deserves further investigation.

Another interesting association could exist between lipid changes and heat shock proteins. In our samples, HSP70 was decreased, while HSP27 was increased after TBI (Tables 1 and 2). The same pattern for these two proteins was detected in white matter plaques from multiple sclerosis patients [87]. Furthermore, it was shown that HSP70 could directly bind MBP, both *in vitro* and in autopsy white matter tissue from multiple sclerosis patients [88], which also parallels our observation of both proteins being decreased in optic nerve tissue after TBI. Finally, several studies indicate that extracellular HSP70 can interact with lipids, reviewed in Tytell 2005 [89].

The present work carries some limitations that should be noted. This study explored a single time point after injury and it is possible that some of the biochemical changes observed could be transitory; evaluation of other time points is planned for future investigations. Another option to be explored in the future is changes in neutral lipids, which are present in the optic nerve [90, 91]. They include cholesterol, cholesterol esters, ceramides, free fatty acids, diglycerides, monoglycerides, cerebrosides, etc. Ceramides and other sphingolipids are involved in inflammatory and autoimmune eye diseases, including symptoms of demyelination of the optic nerve [92]. Cholesterol and cholesterol esters are a major component of myelin, including in the optic nerve [90], and changes in their quantities can give an indication of myelin pathology. Furthermore, the observed proteomic and lipidomic changes using the methods described in this work are detected based on analysis of tissue from the full length of the optic nerve, whereas the neuropathological results indicate a more localized area of demyelination. Thus, a combination of lipidomic analysis and imaging (e.g. MALDI imaging) would provide a more localized information about specific changes in lipid classes or individual species within the time course of degeneration and is currently under development.

## Conclusions

In this study, we characterized the neuropathological, proteomic and lipidomic profiles of optic nerve after repeated mild TBI and found good correlation between the three main aspects of tissue changes at 3 weeks post injury. Collectively, these data indicate that TBI has a specific and significant effect on the morphology and biochemistry of the optic nerve, consistent with an ongoing process of axonal damage and myelin breakdown. As visual function analysis was carried out in this model at a later, 3 month time point, we have already demonstrated that the changes identified are early stages in that process and may represent therapeutic targets to block later dysfunction. Further examination of the neuropathological, proteomic, and lipidomic changes would be helpful not only for further elucidation of the pathogenic mechanisms involved in r-mTBI, but also for identification of specific molecular targets for therapeutic intervention. We are now planning studies to address these deficiencies and to provide increased detail for observed changes including definition of the fatty acid composition of the changing molecular species rather than just their total carbon and double bond numbers.

## Supporting Information

S1 Fig Integration of the identified dysregulated proteins into networks: Network #1—Cellular Assembly and Organization, Cellular Function and Maintenance, Tissue Development. Network was generated by Ingenuity Pathway Analysis (IPA). Twenty molecules were affected and IPA score was 46. Solid lines indicate direct interaction. Dashed lines indicate indirect interactions. Red molecules were up-regulated and green molecules were down-regulated. White molecules were not user specified, but were incorporated into the network through relationships with other molecules. Of particular note were the network hubs centered on MBP, spectrin, neurofilaments and tubulins.
(PDF)

S2 Fig Integration of the identified dysregulated proteins into networks: Network #2 –Cancer, Lipid Metabolism, Molecular Transport. Fifteen molecules were affected and IPA score was 32. Solid lines indicate direct interaction. Dashed lines indicate indirect interactions. Red molecules were up-regulated and green molecules were down-regulated. The two molecules with additional circles inside the symbols (HDL, LDL) are part of a complex. White molecules were not user specified, but were incorporated into the network through relationships with other molecules. Of particular note were the network hubs centered on albumin, APOE and heat shock proteins.
(PDF)

S3 Fig Integration of the identified dysregulated proteins into networks: Network #3—Hereditary Disorder, Skeletal and Muscular Disorders, Cell Morphology. Twelve molecules were affected and IPA score was 12. Solid lines indicate direct interaction. Dashed lines indicate indirect interactions. Red molecules were up-regulated and green molecules were down-regulated. White molecules were not user specified, but were incorporated into the network through relationships with other molecules. Of particular note were the network hubs centered on COL6A1, MSN, and FLNA.
(PDF)

S4 Fig Integration of the identified dysregulated proteins into networks: Network #4 -Behavior, Cell Morphology, Cellular Function and Maintenance. Six molecules were affected and IPA score was 10. Solid lines indicate direct interaction. Dashed lines indicate indirect interactions. Red molecules were up-regulated and green molecules were down-regulated. White molecules were not user specified, but were incorporated into the network through relationships with other molecules. Of particular note were the network hubs centered on CNNG, QDPR and LAP3.
(PDF)

S1 Table Proteomic data for significantly up- or downregulated proteins at in optic nerve tissue at 3 weeks post injury. Proteins are listed ranked by the level of dysregulation: the down-regulated proteins sorted from the most downregulated to the least downregulated, while the unregulated sorted from the least upregulated to the most upregulated.
(PDF)

S2 Table Canonical pathways associated with the effects of r-mTBI on optic nerve tissue at 3 weeks post injury. The list shows pathways that were significantly upregulated with log(p-value) >1.3 (equivalent to a p-value of <0.05). The radio value represents the number of molecules affected in the current data set for the particular pathway compared to the total number of molecules in that pathway according to the IPA analysis.
(PDF)

S3 Table MS/MS product ion identification of fatty acid composition and position for major molecular species detected. (PDF)

S4 Table Phospholipid molecular species identified in optic nerve tissue at 3 weeks post injury by LC/MS. Mass estimate (m/z) for each species was obtained either in positive ion mode [M+H]^+^ or in negative ion mode [M-H]^-^. Mean values and standard deviations are expressed in μg/sample. Significant change is calculated by t-test and the level of significance indicated with asterisks (*—p<0.05, **—p<0.01, ***—p<0.001).
(PDF)
