# Supplementary material for: Sub-Chronic Neuropathological and Biochemical Changes in Mouse Visual System after Repetitive Mild Traumatic Brain Injury
Source: PLoS One. 2016 Apr 18;11(4):e0153608. doi: 10.1371/journal.pone.0153608 (PMC4835061; doi:10.1371/journal.pone.0153608)

S1 Figure. Integration of the identified dysregulated proteins into networks: Network #1 - Cellular Assembly and Organization, Cellular Function and Maintenance, Tissue Development. Network was generated by Ingenuity Pathway Analysis (IPA). Twenty molecules were affected and IPA score was 46. Solid lines indicate direct interaction. Dashed lines indicate indirect interactions. Red molecules were up-regulated and green molecules were down-regulated. White molecules were not user specified, but were incorporated into the network through relationships with other molecules. Of particular note were the network hubs centered on MBP, spectrin, neurofilaments and tubulins.

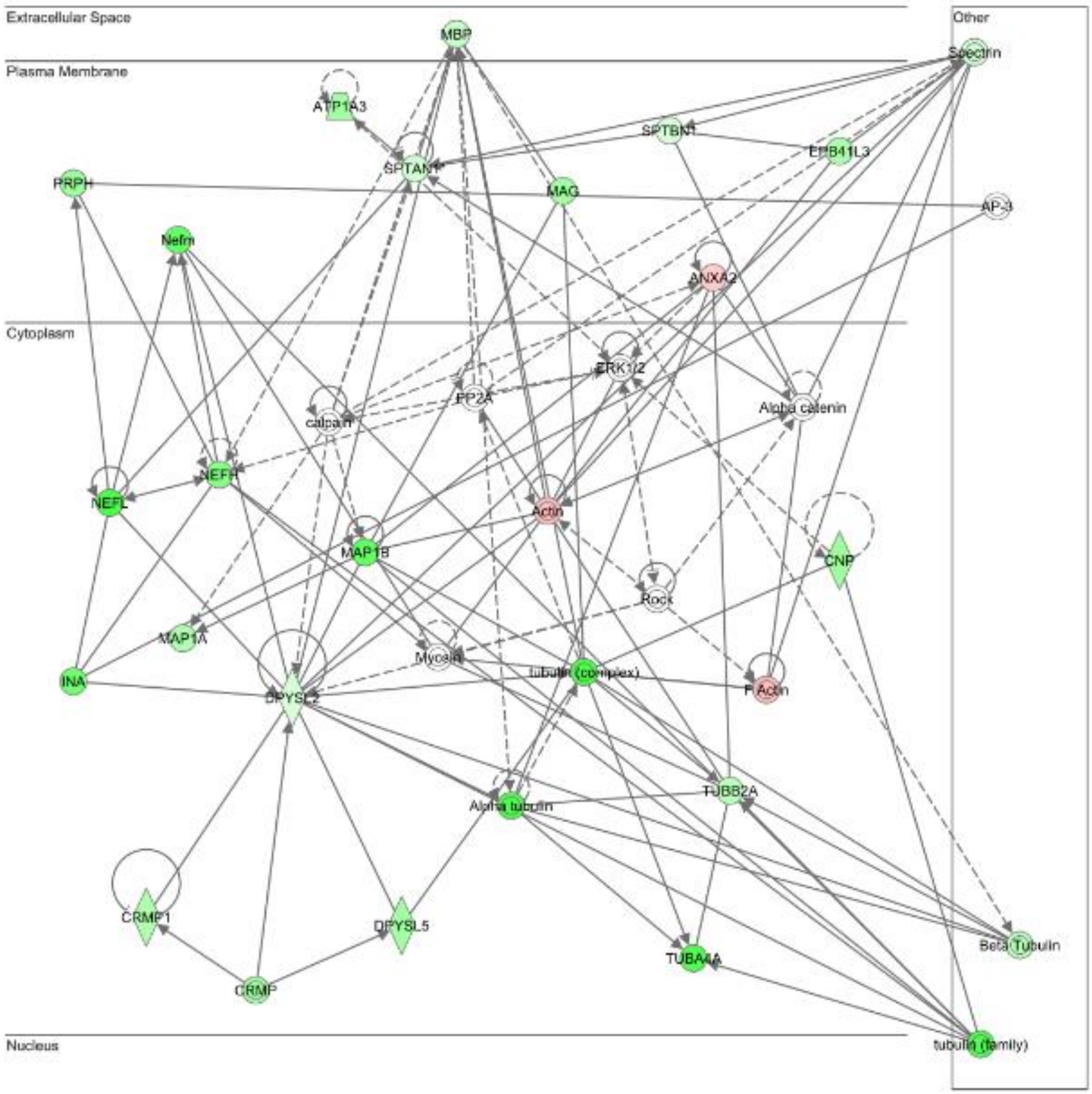

Supplement: S1 Fig — Network was generated by Ingenuity Pathway Analysis (IPA). Twenty molecules were affected and IPA score was 46. Solid lines indicate direct interaction. Dashed lines indicate indirect interactions. Red molecules were up-regulated and green molecules were down-regulated. White molecules were not user specified, but were incorporated into the network through relationships with other molecules. Of particular note were the network hubs centered on MBP, spectrin, neurofilaments and tubulins. (PDF) [file pone.0153608.s001.pdf]
