# Supplementary material for: Sub-Chronic Neuropathological and Biochemical Changes in Mouse Visual System after Repetitive Mild Traumatic Brain Injury
Source: PLoS One. 2016 Apr 18;11(4):e0153608. doi: 10.1371/journal.pone.0153608 (PMC4835061; doi:10.1371/journal.pone.0153608)

S2 Fig. Integration of the identified dysregulated proteins into networks: Network #2 – **Cancer, Lipid Metabolism, Molecular Transport**. Fifteen molecules were affected and IPA score was 32. Solid lines indicate direct interaction. Dashed lines indicate indirect interactions. Red molecules were up-regulated and green molecules were down-regulated. The two molecules with additional circles inside the symbols (HDL, LDL) are part of a complex. White molecules were not user specified, but were incorporated into the network through relationships with other molecules. Of particular note were the network hubs centered on albumin, APOE and heat shock proteins.

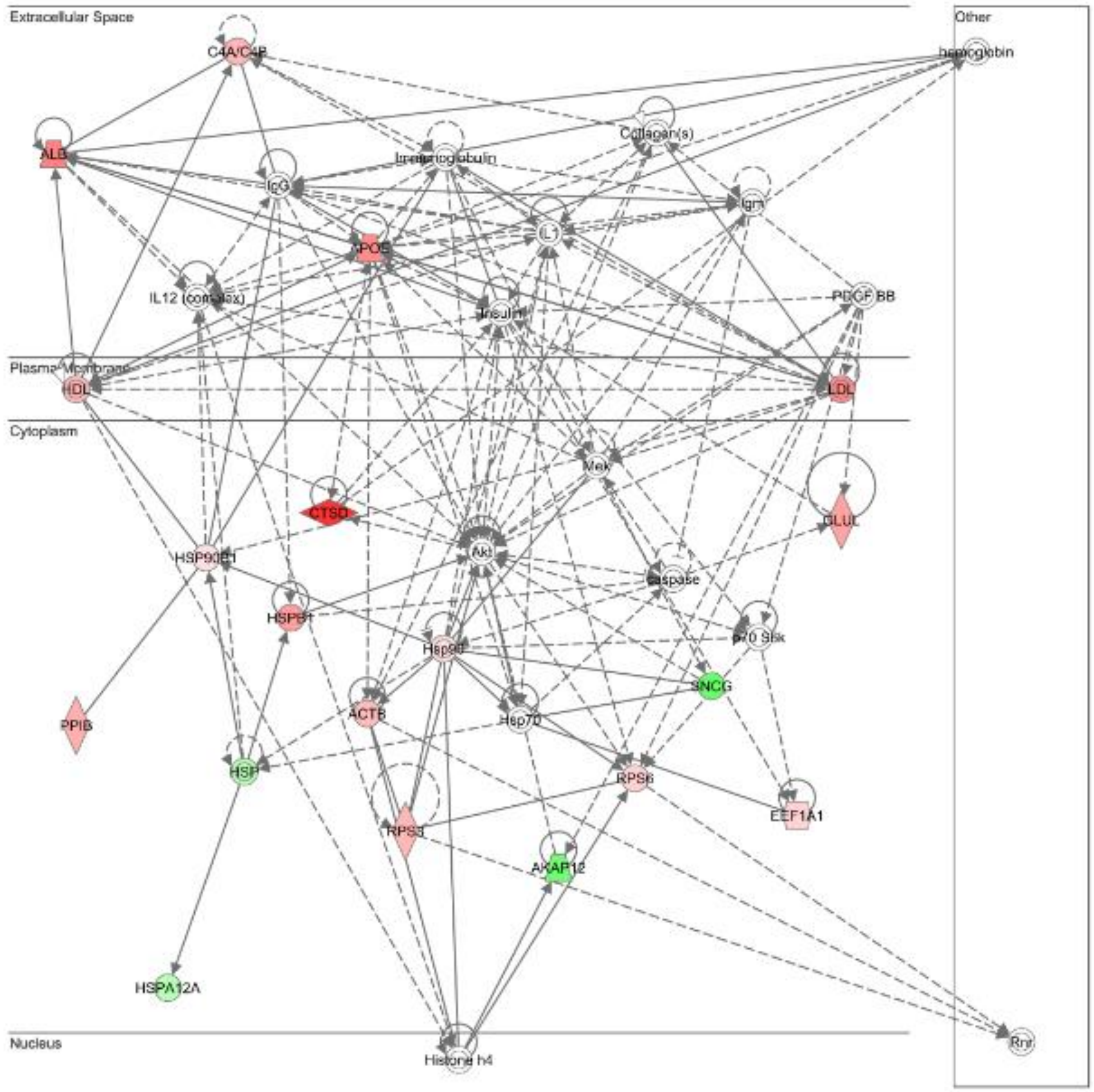

Supplement: S2 Fig — Fifteen molecules were affected and IPA score was 32. Solid lines indicate direct interaction. Dashed lines indicate indirect interactions. Red molecules were up-regulated and green molecules were down-regulated. The two molecules with additional circles inside the symbols (HDL, LDL) are part of a complex. White molecules were not user specified, but were incorporated into the network through relationships with other molecules. Of particular note were the network hubs centered on albumin, APOE and heat shock proteins. (PDF) [file pone.0153608.s002.pdf]
