# Supplementary material for: Sub-Chronic Neuropathological and Biochemical Changes in Mouse Visual System after Repetitive Mild Traumatic Brain Injury
Source: PLoS One. 2016 Apr 18;11(4):e0153608. doi: 10.1371/journal.pone.0153608 (PMC4835061; doi:10.1371/journal.pone.0153608)

S3 Figure. Integration of the identified dysregulated proteins into networks: Network #3 - Hereditary Disorder, Skeletal and Muscular Disorders, Cell Morphology. Twelve molecules were affected and IPA score was 12. Solid lines indicate direct interaction. Dashed lines indicate indirect interactions. Red molecules were up-regulated and green molecules were down-regulated. White molecules were not user specified, but were incorporated into the network through relationships with other molecules. Of particular note were the network hubs centered on COL6A1, MSN, and FLNA.

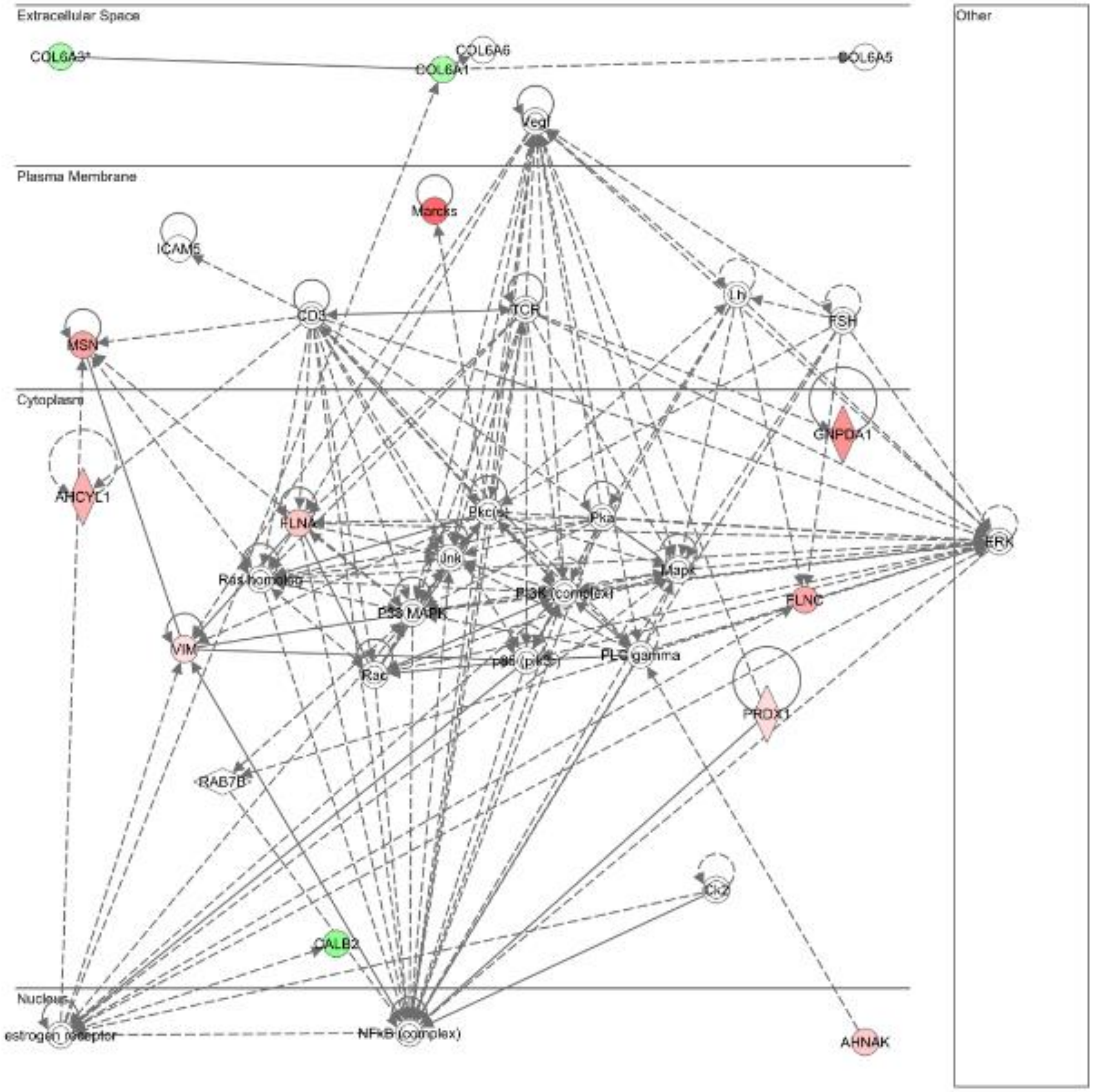

Supplement: S3 Fig — Twelve molecules were affected and IPA score was 12. Solid lines indicate direct interaction. Dashed lines indicate indirect interactions. Red molecules were up-regulated and green molecules were down-regulated. White molecules were not user specified, but were incorporated into the network through relationships with other molecules. Of particular note were the network hubs centered on COL6A1, MSN, and FLNA. (PDF) [file pone.0153608.s003.pdf]
