# Supplementary material for: Sub-Chronic Neuropathological and Biochemical Changes in Mouse Visual System after Repetitive Mild Traumatic Brain Injury
Source: PLoS One. 2016 Apr 18;11(4):e0153608. doi: 10.1371/journal.pone.0153608 (PMC4835061; doi:10.1371/journal.pone.0153608)
Supplement: S1 Table — Proteins are listed ranked by the level of dysregulation: the down-regulated proteins sorted from the most downregulated to the least downregulated, while the unregulated sorted from the least upregulated to the most upregulated. (PDF) [file pone.0153608.s005.pdf]

S1 Table. Proteomic data for significantly up- or downregulated proteins at in optic nerve tissue at 3 weeks post injury. Proteins are listed ranked by the level of dysregulation: the down-regulated proteins sorted from the most downregulated to the least downregulated, while the unregulated sorted from the least upregulated to the most upregulated.

Accession	Description	DF	Mean	SD	t Test	# AAs	MW [kDa]	Σ# PSMs_10-20%ACN	Σ# PSMs_30-40%ACN	Σ# Unique Peptides_10-20%ACN	Σ# Unique Peptides_30-40%ACN	ΣCoverage_10-20%ACN	ΣCoverage_30-40%ACN	Score SEQUEST_10-20%ACN	Score SEQUEST_30-40%ACN	Score Byonic_10-20%ACN	Score Byonic_30-40%ACN	Σ# Peptides_10-20%ACN	Σ# Peptides_30-40%ACN	Σ# Proteins_10-20%ACN	Σ# Proteins_30-40%ACN
	**Downregulated proteins**																				
E9PYB0	Protein Ahnak2 (Fragment) OS=Mus musculus GN=Ahnak2 PE=2 SV=1 - [E9PYB0_MOUSE]	11	-0.6257	0.3176	0.00003	1739	182.6	2	5	2	3	5.23	8.11		5.5	4.59	10.27	2	3	1	3
P08551	Neurofilament light polypeptide OS=Mus musculus GN=Nefl PE=1 SV=5 - [NFL_MOUSE]	68	-0.5059	0.3206	0.00000	543	61.5	40	19	17	6	30.94	11.23	75.59	26.34	91.81	31.98	20	7	1	1
P68368	Tubulin alpha-4A chain OS=Mus musculus GN=Tuba4a PE=1 SV=1 - [TBA4A_MOUSE]	11	-0.4962	0.3034	0.00015	448	49.9	14	21	2	2	17.86	21.65	37.38	56.57	15.15	8.92	8	8	2	2
P14873	Microtubule-associated protein 1B OS=Mus musculus GN=Map1b PE=1 SV=2 - [MAP1B_MOUSE]	32	-0.4362	0.2979	0.00000	2464	270.1	8	15	5	7	1.79	3.25	6.51	16.79	20.42	30.62	5	8	1	1
P08553	Neurofilament medium polypeptide OS=Mus musculus GN=Nefm PE=1 SV=4 - [NFM_MOUSE]	86	-0.4262	0.3369	0.00000	848	95.9	65	31	26	12	30.78	14.98	95.46	38.54	170.37	63.15	29	13	2	2
Q9Z0F7	Gamma-synuclein OS=Mus musculus GN=Sncg PE=1 SV=1 - [SYUG_MOUSE]	8	-0.4165	0.1846	0.00014	123	13.2	6		3		21.95		9.28		12.05		3		2	
Q9WTQ5-2	Isoform 2 of A-kinase anchor protein 12 OS=Mus musculus GN=Akap12 - [AKA12_MOUSE]	5	-0.3996	0.1828	0.00305	1579	169.6		3		2		0.95		2.46		5.53		2		2
P46660	Alpha-internexin OS=Mus musculus GN=Ina PE=1 SV=3 - [AINX_MOUSE]	53	-0.3989	0.3141	0.00000	501	55.3	31	21	13	8	30.74	17.17	61.94	23.07	80.43	39.79	16	9	1	1
P19246	Neurofilament heavy polypeptide OS=Mus musculus GN=Nefh PE=1 SV=3 - [NFH_MOUSE]	76	-0.3301	0.5159	0.00000	1090	116.9	38	12	19	7	28.17	5.87	59.49	12.65	107.26	22.02	20	7	1	1
Q08331	Calretinin OS=Mus musculus GN=Calb2 PE=1 SV=3 - [CALB2_MOUSE]	35	-0.3053	0.3202	0.00000	271	31.4	15	18	6	5	21.77	16.97	18.83	21.78	44.34	39.14	6	5	1	2
P15331-3	Isoform 5b of Peripherin OS=Mus musculus GN=Prph - [PERI_MOUSE]	5	-0.2747	0.1107	0.00174	461	52.7	7	3	1	1	8.24	4.56	19.05	4.28	1.77	1.83	5	2	7	6
P16330	2',3'-cyclic-nucleotide 3'-phosphodiesterase OS=Mus musculus GN=Cnp PE=1 SV=3 - [CN37_MOUSE]	92	-0.2681	0.3807	0.00000	420	47.1	40	49	15	21	26.19	35.95	55.29	54.98	102.73	89.68	15	22	2	17
Q9EQF6	Dihydropyrimidinase-related protein 5 OS=Mus musculus GN=Dpysl5 PE=1 SV=1 - [DPYL5_MOUSE]	20	-0.2602	0.3036	0.00083	564	61.5	3	14	2	5	2.48	7.62	2.43	13.32	3.68	22.55	2	5	2	2
Q6PIC6	Sodium/potassium-transporting ATPase subunit alpha-3 OS=Mus musculus GN=Atp1a3 PE=1 SV=1 - [AT	35	-0.2592	0.3009	0.00001	1013	111.6	35	53	8	6	15.5	16.98	63.32	70.39	102.79	115.55	14	16	5	7
P20917-2	Isoform S-MAG of Myelin-associated glycoprotein OS=Mus musculus GN=Mag - [MAG_MOUSE]	14	-0.2553	0.2819	0.00348	582	64.2	6		5		6.19		4.13		19.68		5		2	
J3QQ16	Protein Col6a3 OS=Mus musculus GN=Col6a3 PE=2 SV=1 - [J3QQ16_MOUSE]	38	-0.2510	0.3209	0.00002	2677	288.5		28		15		5.01		30.61		53.37		15		3
Q9WV92-7	Isoform 7 of Band 4.1-like protein 3 OS=Mus musculus GN=Epb41l3 - [E41L3_MOUSE]	20	-0.2373	0.2095	0.00004	823	91.8	3	17	2	5	2.07	9.36	2.41	13.51	9.02	23.83	2	8	8	16
Q04857	Collagen alpha-1(VI) chain OS=Mus musculus GN=Col6a1 PE=1 SV=1 - [CO6A1_MOUSE]	35	-0.2275	0.2767	0.00002	1025	108.4	11	13	7	7	5.76	6.63	10.34	18.33	23.94	38.94	7	7	1	1
A2ARP8	Microtubule-associated protein 1A OS=Mus musculus GN=Map1a PE=2 SV=1 - [A2ARP8_MOUSE]	42	-0.2249	0.2810	0.00000	3014	325.7	12	25	7	10	2.29	3.45	14.54	24.63	38.09	43.29	7	11	3	3
P97427	Dihydropyrimidinase-related protein 1 OS=Mus musculus GN=Crmp1 PE=1 SV=1 - [DPYL1_MOUSE]	14	-0.2232	0.2287	0.00203	572	62.1	4	8	2	3	5.59	6.82	8.2	17	6.59	13.83	3	4	2	2
E9PWQ3	Protein Col6a3 OS=Mus musculus GN=Col6a3 PE=2 SV=2 - [E9PWQ3_MOUSE]	29	-0.2100	0.2888	0.00042	3284	353.7	14		10		2.8		11.56		37.84		10		3	
Q8K0U4	Heat shock 70 kDa protein 12A OS=Mus musculus GN=Hspa12a PE=1 SV=1 - [HS12A_MOUSE]	35	-0.1973	0.2608	0.00006	675	74.8	9	19	5	10	7.7	15.26	11.59	26.55	26.19	41.51	5	10	1	2
Q7TMM9	Tubulin beta-2A chain OS=Mus musculus GN=Tubb2a PE=1 SV=1 - [TBB2A_MOUSE]	23	-0.1911	0.2878	0.00351	445	49.9	14	34	1	3	22.7	25.39	50.27	68.58	3.7	39.99	8	10	3	3
P04370-9	Isoform 9 of Myelin basic protein OS=Mus musculus GN=Mbp - [MBP_MOUSE]	44	-0.1488	0.3139	0.00270	184	20.2	77		13		57.61		106.91		97.49		13		18	
A3KGU5	Spectrin alpha chain, non-erythrocytic 1 OS=Mus musculus GN=Sptan1 PE=2 SV=1 - [A3KGU5_MOUSE]	102	-0.1402	0.3107	0.00001	2457	282.7		70		33		11.44		86.69		147.97		33		6
Q62261	Spectrin beta chain, non-erythrocytic 1 OS=Mus musculus GN=Sptbn1 PE=1 SV=2 - [SPTB2_MOUSE]	95	-0.1092	0.2165	0.00000	2363	274.1	35	32	20	15	7.66	6.26	45.79	43	109.31	82.95	20	15	3	7
O08553	Dihydropyrimidinase-related protein 2 OS=Mus musculus GN=Dpysl2 PE=1 SV=2 - [DPYL2_MOUSE]	56	-0.1069	0.2480	0.00192	572	62.2	30	35	13	9	32.52	21.85	52.53	47.31	109.2	76.91	14	11	1	1
P16546	Spectrin alpha chain, non-erythrocytic 1 OS=Mus musculus GN=Sptan1 PE=1 SV=4 - [SPTN1_MOUSE]	79	-0.0955	0.2183	0.00019	2472	284.4	47		26		9.95		57.92		136.38		26		7	
	**Upnregulated proteins**																				
P08113	Endoplasmin OS=Mus musculus GN=Hsp90b1 PE=1 SV=2 - [ENPL_MOUSE]	32	0.0816	0.1453	0.00289	802	92.4	8	10	5	6	5.74	5.86	9.39	10.49	23.02	19.2	5	6	2	2
P20152	Vimentin OS=Mus musculus GN=Vim PE=1 SV=3 - [VIME_MOUSE]	110	0.0905	0.2437	0.00016	466	53.7	101	48	25	14	56.65	30.04	156.83	50.52	276.37	87.97	32	16	5	22
P35700	Peroxiredoxin-1 OS=Mus musculus GN=Prdx1 PE=1 SV=1 - [PRDX1_MOUSE]	35	0.1064	0.1799	0.00113	199	22.2	18	28	7	9	32.16	45.23	26.35	40.94	47.65	54.75	7	9	6	6
P10126	Elongation factor 1-alpha 1 OS=Mus musculus GN=Eef1a1 PE=1 SV=3 - [EF1A1_MOUSE]	26	0.1269	0.2153	0.00504	462	50.1	8	17	5	7	8.66	12.99	7.54	19.87	19.21	31.56	5	7	4	4
P62754	40S ribosomal protein S6 OS=Mus musculus GN=Rps6 PE=1 SV=1 - [RS6_MOUSE]	8	0.1335	0.1046	0.00503	249	28.7	4	1	2	1	8.03	2.01	5.36		10.31	2.83	2	1	1	1
E9Q616	Protein Ahnak OS=Mus musculus GN=Ahnak PE=2 SV=1 - [E9Q616_MOUSE]	38	0.1538	0.1746	0.00000	5656	603.9	7	17	1	10	2.86	5.94	6.25	11.35	11.98	34.01	2	10	1	1
P07356	Annexin A2 OS=Mus musculus GN=Anxa2 PE=1 SV=2 - [ANXA2_MOUSE]	31	0.1547	0.2146	0.00030	339	38.7	12	6	7	4	20.65	10.62	19.35	5.75	40.2	15.27	7	4	4	4
B7FAV1	Filamin, alpha (Fragment) OS=Mus musculus GN=Flna PE=4 SV=1 - [B7FAV1_MOUSE]	5	0.1672	0.0832	0.00438	2583	274.5	4		2		0.85		5.37		5.91		3		4	
Q8C0M9	Isoaspartyl peptidase/L-asparaginase OS=Mus musculus GN=Asrgl1 PE=1 SV=1 - [ASGL1_MOUSE]	17	0.1698	0.1895	0.00143	326	33.9	5	6	3	3	7.98	10.74	4.42	9.07	8.92	14.58	3	3	1	1
Q9CPY7-2	Isoform 2 of Cytosol aminopeptidase OS=Mus musculus GN=Lap3 - [AMPL_MOUSE]	14	0.1873	0.1880	0.00173	488	52.7	3	6	2	3	3.28	6.35	1.64	8.87	3.81	16.41	2	3	2	2
P60710	Actin, cytoplasmic 1 OS=Mus musculus GN=Actb PE=1 SV=1 - [ACTB_MOUSE]	53	0.1941	0.2052	0.00000	375	41.7	38	47	5	9	34.93	24.27	71.16	58.47	120.1	78.33	10	10	11	18
P01029	Complement C4-B OS=Mus musculus GN=C4b PE=1 SV=3 - [CO4B_MOUSE]	11	0.2085	0.1237	0.00011	1738	192.8	2	6	1	3	0.4	1.5	1.91	8.25	1.86	10.07	1	3	1	1
P62908	40S ribosomal protein S3 OS=Mus musculus GN=Rps3 PE=1 SV=1 - [RS3_MOUSE]	14	0.2164	0.2317	0.00280	243	26.7		9		5		18.11		12.67		17.64		5		2
Q80SW1	Putative adenosylhomocysteinase 2 OS=Mus musculus GN=Ahcyl1 PE=1 SV=1 - [SAHH2_MOUSE]	5	0.2340	0.1100	0.00343	530	58.9		4		2		4.72		4.96		7.5		3		1
P24369	Peptidyl-prolyl cis-trans isomerase B OS=Mus musculus GN=Ppib PE=2 SV=2 - [PPIB_MOUSE]	8	0.2341	0.1351	0.00082	216	23.7	3	1	2	1	6.02	3.24	1.81	1.94	5.65		2	1	1	1
Q8VHX6-2	Isoform 2 of Filamin-C OS=Mus musculus GN=Flnc - [FLNC_MOUSE]	41	0.2544	0.2231	0.00000	2693	287.2	5	25	2	11	1.15	4.75	8.24	31.73	12.3	56.09	3	12	4	5
Q8BVI4	Dihydropteridine reductase OS=Mus musculus GN=Qdpr PE=1 SV=2 - [DHPR_MOUSE]	17	0.2566	0.1883	0.00002	241	25.6	4	10	2	5	6.22	22.82	3.84	13.73	8.05	27.92	2	5	4	3
P15105	Glutamine synthetase OS=Mus musculus GN=Glul PE=1 SV=6 - [GLNA_MOUSE]	23	0.2759	0.1317	0.00000	373	42.1	8	8	5	4	12.33	11.8	11.54	9.54	25.67	15.36	5	4	2	3
P26041	Moesin OS=Mus musculus GN=Msn PE=1 SV=3 - [MOES_MOUSE]	17	0.2759	0.2076	0.00003	577	67.7	13	6	4	2	10.57	5.55	17.08	0	32.11	10.54	7	4	3	3
D3YZ06	Heat shock protein beta-1 OS=Mus musculus GN=Hspb1 PE=2 SV=1 - [D3YZ06_MOUSE]	5	0.2870	0.0509	0.00004	147	16.8		4		2		17.69		7.52		13.22		2		4
Q8CGP1	Histone H2B type 1-K OS=Mus musculus GN=Hist1h2bk PE=1 SV=3 - [H2B1K_MOUSE]	17	0.2932	0.1830	0.00000	126	13.9	12		6		37.3		13.37		31.24		6		15	
O88958	Glucosamine-6-phosphate isomerase 1 OS=Mus musculus GN=Gnpda1 PE=2 SV=3 - [GNPI1_MOUSE]	5	0.3240	0.1466	0.00291	289	32.5		3		2		5.19		2.78		6.07		2		1
P08226	Apolipoprotein E OS=Mus musculus GN=Apoe PE=1 SV=2 - [APOE_MOUSE]	14	0.3418	0.3220	0.00106	311	35.8	9	2	5	1	17.36	2.89	11.12	3.11	21.02	6.1	5	1	4	1
A8DUK4	Beta-globin OS=Mus musculus GN=Hbbt1 PE=3 SV=1 - [A8DUK4_MOUSE]	26	0.3724	0.3870	0.00003	147	15.7	16	35	4	7	29.25	49.66	21.02	52.77	29.54	61.73	4	7	4	5
P07724	Serum albumin OS=Mus musculus GN=Alb PE=1 SV=3 - [ALBU_MOUSE]	59	0.3783	0.2760	0.00000	608	68.6	21	32	11	14	16.45	21.38	22.99	43.95	45.68	66.23	11	14	1	2
F6QL70	Protein Gm17669 OS=Mus musculus GN=Gm17669 PE=4 SV=1 - [F6QL70_MOUSE]	5	0.3931	0.1540	0.00153	154	17	4		2		11.69		3.19		6.28		2		4	
Q9DAW9	Calponin-3 OS=Mus musculus GN=Cnn3 PE=1 SV=1 - [CNN3_MOUSE]	11	0.4011	0.2519	0.00018	330	36.4	7		4		11.82		8.6		19.81		4		3	
P26645	Myristoylated alanine-rich C-kinase substrate OS=Mus musculus GN=Marcks PE=1 SV=2 - [MARCS_MOU	5	0.4502	0.1946	0.00238	309	29.6	4	2	1	1	6.15	2.27	7.78	2.38	10.02	2.54	1	1	1	1
P18242	Cathepsin D OS=Mus musculus GN=Ctsd PE=1 SV=1 - [CATD_MOUSE]	14	0.6162	0.6666	0.00301	410	44.9	1	13	1	5	1.95	9.02		8.08	2.72	20.48	1	5	1	3

Legend: DF – degrees of freedom, SD – standard deviation, #AAs – number of amino acids, MW –molecular weight, PSMs - peptide–spectrum matches, ACN – acetonitrile
