# Supplementary material for: Sub-Chronic Neuropathological and Biochemical Changes in Mouse Visual System after Repetitive Mild Traumatic Brain Injury
Source: PLoS One. 2016 Apr 18;11(4):e0153608. doi: 10.1371/journal.pone.0153608 (PMC4835061; doi:10.1371/journal.pone.0153608)
Supplement: S2 Table — The list shows pathways that were significantly upregulated with log(p-value) >1.3 (equivalent to a p-value of <0.05). The radio value represents the number of molecules affected in the current data set for the particular pathway compared to the total number of molecules in that pathway according to the IPA analysis. (PDF) [file pone.0153608.s006.pdf]

S2 Table. Canonical pathways associated with the effects of r-mTBI on optic nerve tissue at 3 weeks post injury. The list shows pathways that were significantly upregulated with log(p-value) >1.3 (equivalent to a p-value of <0.05). The radio value represents the number of molecules affected in the current data set for the particular pathway compared to the total number of molecules in that pathway according to the IPA analysis.

#	Ingenuity Canonical Pathways	-log(p-value)	Ratio	# molecules	Molecules
1	Caveolar-mediated Endocytosis Signaling	4.33E00	5.56E-02	4	ALB,FLNA,FLNC,ACTB
2	Sertoli Cell-Sertoli Cell Junction Signaling	3.87E00	2.79E-02	5	SPTBN1,ACTB,TUBB2A,TUBA4A,SPTAN1
3	Amyotrophic Lateral Sclerosis Signaling	3.81E00	4.08E-02	4	PRPH,NEFL,NEFH,GLUL
4	Semaphorin Signaling in Neurons	3.37E00	5.66E-02	3	DPYSL2,CRMP1,DPYSL5
5	Remodeling of Epithelial Adherens Junctions	3.05E00	4.41E-02	3	ACTB,TUBB2A,TUBA4A
6	ILK Signaling	2.76E00	2.15E-02	4	FLNA,FLNC,ACTB,VIM
7	Virus Entry via Endocytic Pathways	2.72E00	3.37E-02	3	FLNA,FLNC,ACTB
8	Death Receptor Signaling	2.68E00	3.26E-02	3	ACTB,SPTAN1,HSPB1
9	Glutamine Biosynthesis I	2.56E00	1E00	1	GLUL
10	14-3-3-mediated Signaling	2.38E00	2.56E-02	3	TUBB2A,TUBA4A,VIM
11	LXR/RXR Activation	2.34E00	2.48E-02	3	C4A/C4B,APOE,ALB
12	FXR/RXR Activation	2.28E00	2.36E-02	3	C4A/C4B,APOE,ALB
13	Asparagine Degradation I	2.26E00	5E-01	1	ASRGL1
14	Axonal Guidance Signaling	2.17E00	1.16E-02	5	DPYSL2,MAG,TUBB2A,TUBA4A,DPYSL5
15	Aryl Hydrocarbon Receptor Signaling	2.16E00	2.14E-02	3	CTSD,HSP90B1,HSPB1
16	Epithelial Adherens Junction Signaling	2.11E00	2.05E-02	3	ACTB,TUBB2A,TUBA4A
17	Thyroid Hormone Biosynthesis	2.08E00	3.33E-01	1	CTSD
18	N-acetylglucosamine Degradation I	2.08E00	3.33E-01	1	GNPDA1
19	Aldosterone Signaling in Epithelial Cells	2.07E00	1.97E-02	3	HSP90B1,HSPA12A,HSPB1
20	Gap Junction Signaling	2.04E00	1.94E-02	3	ACTB,TUBB2A,TUBA4A
21	Germ Cell-Sertoli Cell Junction Signaling	2.01E00	1.88E-02	3	ACTB,TUBB2A,TUBA4A
22	Uracil Degradation II (Reductive)	1.96E00	2.5E-01	1	DPYSL2
23	Thymine Degradation	1.96E00	2.5E-01	1	DPYSL2
24	N-acetylglucosamine Degradation II	1.96E00	2.5E-01	1	GNPDA1
25	Phenylalanine Degradation I (Aerobic)	1.96E00	2.5E-01	1	QDPR
26	NRF2-mediated Oxidative Stress Response	1.87E00	1.67E-02	3	PPIB,PRDX1,ACTB
27	Clathrin-mediated Endocytosis Signaling	1.84E00	1.62E-02	3	APOE,ALB,ACTB
28	Actin Cytoskeleton Signaling	1.65E00	1.38E-02	3	FLNA,ACTB,MSN
29	UDP-N-acetyl-D-galactosamine Biosynthesis II	1.61E00	1.11E-01	1	GNPDA1
30	Neuregulin Signaling	1.61E00	2.27E-02	2	RPS6,HSP90B1
31	Signaling by Rho Family GTPases	1.57E00	1.28E-02	3	ACTB,VIM,MSN
32	Protein Ubiquitination Pathway	1.48E00	1.18E-02	3	HSP90B1,HSPA12A,HSPB1
33	Methionine Degradation I (to Homocysteine)	1.36E00	6.25E-02	1	AHCYL1
34	RhoA Signaling	1.35E00	1.64E-02	1	ACTB,MSN
35	Atherosclerosis Signaling	1.34E00	1.63E-02	1	APOE,ALB
36	Cysteine Biosynthesis III (mammalia)	1.31E00	5.56E-02	1	AHCYL1
