# Supplementary material for: Sub-Chronic Neuropathological and Biochemical Changes in Mouse Visual System after Repetitive Mild Traumatic Brain Injury
Source: PLoS One. 2016 Apr 18;11(4):e0153608. doi: 10.1371/journal.pone.0153608 (PMC4835061; doi:10.1371/journal.pone.0153608)
Supplement: S3 Table — (PDF) [file pone.0153608.s007.pdf]

S3 Table. MS/MS product ion identification of fatty acid composition and position for major molecular species detected. The relative abundance of two fatty acids is shown in the cells highlighted in green.

Precursor	Head Grp	Species	SN1	SN2	%
750.6	PC	30:0	16:0	14:0	
778.6	PC	32:0	16:0	16:0	
778.6	PC	32:0	16:0	16:0	
776.6	PC	32:1	16:0	16:1	
804.6	PC	34:1	16:0	18:1	
804.6	PC	34:1	16:0	18:1	
832.6	PC	36:1	18:0	18:1	
832.6	PC	36:1	18:0	18:1	
830.6	PC	36:2	18:1	18:1	
830.6	PC	36:2	18:1	18:1	
826.6	PC	36:4	16:0	20:4	
860.7	PC	38:1	18:0	20:1	
858.7	PC	38:2	18:1	20:1	
854.7	PC	38:4	18:0	20:4	
854.7	PC	38:4	18:0	20:4	
888.7	PC	40:1	22:0	18:1	
882.7	PC	40:4	18:0	22:4	
878.7	PC	40:6	18:0	22:6	
916.7	PC	42:1	24:0	18:1	
790.6	PC	o-34:1	o-16:0	18:1	50
790.6	PC	o-34:1	o-18:1	16:0	50
818.6	PC	o-36:1	o-18:0	18:0	
744.6	PE	36:1	18:0	18:1	
744.6	PE	36:1	18:0	18:1	
728.6	PE	36:2	18:1	20:1	
766.6	PE	38:4	18:0	20:4	
790.6	PE	40:6	18:0	22:6	
790.6	PE	40:6	18:0	22:6	
700.6	PE	o-34:2	o-16:1	18:1	80
700.6	PE	o-34:2	o-18:2	16:0	20
700.6	PE	o-34:2	o-16:1	18:1	70
700.6	PE	o-34:2	o-18:2	16:0	30
728.6	PE	o-36:2	o-16:1	20:1	30
728.6	PE	o-36:2	o-18:1	18:1	70
726.6	PE	o-36:3	o-18:2	18:1	
756.6	PE	o-38:2	o-18:1	20:1	85
756.6	PE	o-38:2	o-18:2	20:0	15
750.6	PE	o-38:5	o-16:1	22:4	30
750.6	PE	o-38:5	o-18:1	22:5	70
750.6	PE	o-38:5	o-16:1	22:6	40
750.6	PE	o-38:5	o-18:1	22:7	60
778.6	PE	o-40:5	o-18:1	22:4	
774.6	PE	o-40:7	o-18:1	22:6	
747.6	PG	34:1	16:0	18:1	
885.7	PI	38:4	18:0	20:4	
