# Supplementary material for: Sub-Chronic Neuropathological and Biochemical Changes in Mouse Visual System after Repetitive Mild Traumatic Brain Injury
Source: PLoS One. 2016 Apr 18;11(4):e0153608. doi: 10.1371/journal.pone.0153608 (PMC4835061; doi:10.1371/journal.pone.0153608)
Supplement: S4 Table — Mass estimate (m/z) for each species was obtained either in positive ion mode [M+H]+ or in negative ion mode [M-H]-. Mean values and standard deviations are expressed in μg/sample. Significant change is calculated by t-test and the level of significance indicated with asterisks (*—p<0.05, **—p<0.01, ***—p<0.001). (PDF) [file pone.0153608.s008.pdf]

S4 Table. Phospholipid molecular species identified in optic nerve tissue at 3 weeks post injury by LC/MS. Mass estimate (m/z) for each specie was obtained either in positive ion mode [M+H]$^+$ or in negative ion mode [M-H]$^-$**.** Mean values and standard deviations are expressed in g/sample. Significant change is calculated by t-test and the level of significance indicated with asterisks (* - p<0.05, ** - p<0.01, *** - p<0.001).

A. Ether Phosphatydilcholine (ePC)

			r-sham (n=5)			r-mTBI (n = 5)				
	Name	[M+H]$^+$	Mean	SD	% of total	Mean	SD	% of total	% change	Sign. change
1	ePC(32:0)	720.6	0.073	0.022	5.9%	0.101	0.013	7.6%	38.7%	
2	ePC(32:1)	718.6	0.050	0.011	4.1%	0.061	0.009	4.6%	21.2%	
3	ePC(32:2)	716.6	0.018	0.004	1.5%	0.017	0.004	1.3%	-7.0%	
4	ePC(34:0)	748.6	0.075	0.015	6.1%	0.058	0.013	4.4%	-22.6%	
5	ePC(34:2)	744.6	0.080	0.016	6.5%	0.097	0.014	7.3%	21.6%	
6	ePC(36:0)	776.6	0.060	0.019	4.8%	0.064	0.030	4.8%	8.1%	
7	ePC(36:2)	772.6	0.061	0.008	5.0%	0.024	0.018	1.8%	-61.2%	**
8	ePC(36:3)	770.6	0.031	0.007	2.5%	0.039	0.008	2.9%	25.8%	
9	ePC(36:4)	768.6	0.041	0.011	3.3%	0.065	0.013	4.9%	56.9%	*
10	ePC(36:5)	766.6	0.034	0.007	2.8%	0.048	0.009	3.6%	38.2%	*
11	ePC(38:0)	804.7	0.042	0.013	3.4%	0.050	0.016	3.8%	20.2%	
12	ePC(38:1)	802.7	0.147	0.032	11.9%	0.136	0.030	10.2%	-6.9%	
13	ePC(38:2)	800.6	0.097	0.021	7.8%	0.078	0.018	5.9%	-18.9%	
14	ePC(38:3)	798.6	0.077	0.018	6.3%	0.083	0.021	6.2%	7.4%	
15	ePC(38:5)	794.6	0.054	0.016	4.4%	0.098	0.021	7.4%	80.3%	**
16	ePC(38:6)	792.6	0.082	0.023	6.6%	0.097	0.026	7.3%	19.1%	
17	ePC(40:2)	828.7	0.061	0.014	4.9%	0.055	0.014	4.1%	-9.2%	
18	ePC(40:3)	826.7	0.039	0.010	3.2%	0.035	0.010	2.7%	-10.1%	
19	ePC(40:4)	824.6	0.031	0.010	2.5%	0.034	0.010	2.5%	9.0%	
20	ePC(40:5)	822.6	0.033	0.009	2.7%	0.034	0.010	2.6%	3.7%	
21	ePC(40:6)	820.6	0.048	0.016	3.9%	0.056	0.016	4.2%	18.5%	
		TOTAL ePC	1.234			1.332				

B. Ether Lysophosphatydilcholine (eLPC)

			r-sham (n=5)			r-mTBI (n = 5)				
	Name	[M+H]$^+$	Mean	SD	% of total	Mean	SD	% of total	% change	sign. change
1	eLPC(16:1)	480.3	0.013	0.012	0.009	0.016	0.003	12.5%	23.1%	**
2	eLPC(16:0)	482.4	0.019	0.02	0.017	0.047	0.001	36.4%	146.1%	***
3	eLPC(18:1)	508.4	0.014	0.011	0.016	0.018	0.003	13.8%	26.8%	*
4	eLPC(18:0)	510.4	0.016	0.017	0.016	0.048	0.006	37.4%	200.0%	***
		TOTAL eLPC	0.062			0.129				***

C. Ether Phosphatydilethanolamine (ePE)

	Name	[M-H]-	r-sham (n=5) Mean	SD	% of total	r-mTBI (n = 5) Mean	SD	% of total	% change	sign. change
1	ePE(32:1)	674.5	0.937	0.286	3.7%	0.711	0.296	3.5%	-24.1%	
2	ePE(32:2)	672.5	0.095	0.024	0.4%	0.121	0.044	0.6%	27.0%	
3	ePE(32:3)	670.5	0.041	0.009	0.2%	0.034	0.007	0.2%	-16.8%	
4	ePE(34:1)	702.6	1.023	0.265	4.0%	0.725	0.286	3.6%	-29.1%	
5	ePE(34:2)	700.5	2.442	0.488	9.6%	1.932	0.564	9.5%	-20.9%	
6	ePE(34:3)	698.5	0.149	0.035	0.6%	0.139	0.041	0.7%	-6.8%	
7	ePE(34:4)	696.5	0.184	0.042	0.7%	0.147	0.043	0.7%	-20.3%	
8	ePE(36:0)	732.6	0.080	0.022	0.3%	0.060	0.008	0.3%	-24.5%	
9	ePE(36:1)	730.6	0.416	0.076	1.6%	0.310	0.104	1.5%	-25.5%	
10	ePE(36:2)	728.6	3.030	0.472	11.9%	2.372	0.745	11.7%	-21.7%	
11	ePE(36:3)	726.6	2.828	0.455	11.1%	2.434	0.726	12.0%	-14.0%	
12	ePE(36:4)	724.5	0.599	0.121	2.4%	0.448	0.145	2.2%	-25.1%	
13	ePE(36:5)	722.5	0.434	0.071	1.7%	0.424	0.109	2.1%	-2.4%	
14	ePE(38:0)	760.6	0.130	0.020	0.5%	0.138	0.027	0.7%	6.4%	
15	ePE(38:1)	758.6	0.315	0.058	1.2%	0.259	0.064	1.3%	-17.6%	
16	ePE(38:2)	756.6	1.566	0.228	6.2%	1.198	0.346	5.9%	-23.5%	
17	ePE(38:3)	754.6	1.837	0.306	7.2%	1.468	0.466	7.2%	-20.1%	
18	ePE(38:4)	752.6	0.830	0.126	3.3%	0.615	0.226	3.0%	-25.9%	
19	ePE(38:5)	750.6	2.039	0.325	8.0%	1.679	0.620	8.3%	-17.6%	
20	ePE(38:6)	748.5	1.289	0.181	5.1%	1.045	0.376	5.1%	-18.9%	
21	ePE(40:2)	784.6	0.302	0.053	1.2%	0.258	0.079	1.3%	-14.4%	
22	ePE(40:3)	782.6	0.514	0.081	2.0%	0.420	0.125	2.1%	-18.2%	
23	ePE(40:4)	780.6	0.485	0.080	1.9%	0.428	0.159	2.1%	-11.7%	
24	ePE(40:5)	778.6	1.859	0.296	7.3%	1.431	0.582	7.0%	-23.0%	
25	ePE(40:6)	776.6	1.968	0.308	7.8%	1.551	0.644	7.6%	-21.2%	
		TOTAL ePE	25.4			20.349				

D. ether Lysophosphatydilethanolamine (eLPE)

N	Name	[M-H]-	r-sham (n=5) Mean	SD	% of total	r-mTBI (n = 5) Mean	SD	% of total	% change	sign. change
1	eLPE(16:0)	438.3	0.014	0.004	3.0%	0.016	0.007	2.4%	20.8%	
2	eLPE(16:1)	436.3	0.048	0.013	10.6%	0.078	0.010	11.5%	59.9%	**
3	eLPE(16:2)	434.3	0.013	0.004	2.9%	0.012	0.005	1.8%	-11.3%	
4	eLPE(18:0)	466.3	0.016	0.002	3.5%	0.026	0.008	3.8%	61.8%	*
5	eLPE(18:1)	464.3	0.097	0.025	21.1%	0.164	0.053	24.4%	69.8%	**
6	eLPE(18:2)	462.3	0.147	0.023	32.1%	0.239	0.068	35.6%	62.3%	**
7	eLPE(20:0)	494.4	0.066	0.018	14.4%	0.062	0.016	9.2%	-6.2%	
9	eLPE(20:2)	490.3	0.057	0.022	12.5%	0.076	0.034	11.3%	32.0%	
		TOTAL eLPE	0.458			0.672				*

E. Phosphatydilcholine (PC)

N	Name	[M+H]+	r-sham (n=5) Mean	r-sham (n=5) SD	r-sham (n=5) % of total	r-mTBI (n = 4) Mean	r-mTBI (n = 4) SD	r-mTBI (n = 4) % of total	% change	Sign. change
1	PC(30:0)	706.5	0.392	0.090	1.3%	0.374	0.088	1.6%	-4.7%	
2	PC(32:0)	734.6	2.841	0.850	9.3%	1.901	0.590	8.0%	-33.1%	
3	PC(32:1)	732.5	0.357	0.119	1.2%	0.235	0.193	1.0%	-34.2%	
4	PC(32:2)	730.5	0.012	0.003	0.0%	0.014	0.004	0.1%	18.8%	
5	PC(34:0)	762.6	0.966	0.213	3.2%	0.497	0.292	2.1%	-48.6%	**
6	PC(34:1)	760.6	10.191	1.891	33.5%	8.028	2.315	33.8%	-21.2%	
7	PC(34:2)	758.6	0.317	0.072	1.0%	0.181	0.079	0.8%	-42.8%	*
8	PC(34:3)	756.5	0.054	0.016	0.2%	0.029	0.011	0.1%	-46.3%	*
9	PC(34:4)	754.5	0.008	0.003	0.0%	0.009	0.004	0.0%	12.5%	
10	PC(36:0)	790.6	0.367	0.097	1.2%	0.291	0.104	1.2%	-20.8%	
11	PC(36:1)	788.6	6.644	1.416	21.8%	5.180	1.493	21.8%	-22.0%	
12	PC(36:3)	784.6	0.196	0.039	0.6%	0.150	0.040	0.6%	-23.6%	
13	PC(36:4)	782.6	0.950	0.193	3.1%	1.145	0.220	4.8%	20.5%	
14	PC(36:5)	780.5	0.021	0.004	0.1%	0.032	0.008	0.1%	50.0%	*
15	PC(38:0)	818.7	0.082	0.023	0.3%	0.086	0.020	0.4%	4.9%	
16	PC(38:1)	816.6	0.726	0.152	2.4%	0.550	0.175	2.3%	-24.3%	
17	PC(38:2)	814.6	0.465	0.085	1.5%	0.298	0.129	1.3%	-36.0%	*
18	PC(38:3)	812.6	0.287	0.058	0.9%	0.221	0.078	0.9%	-23.2%	
19	PC(38:4)	810.6	1.564	0.371	5.1%	1.342	0.357	5.7%	-14.2%	
20	PC(38:5)	808.6	0.478	0.094	1.6%	0.401	0.114	1.7%	-16.1%	
21	PC(38:6)	806.6	0.576	0.131	1.9%	0.513	0.133	2.2%	-10.9%	
22	PC(40:2)	842.7	0.217	0.051	0.7%	0.170	0.057	0.7%	-21.8%	
23	PC(40:3)	840.6	0.111	0.027	0.4%	0.097	0.035	0.4%	-12.8%	
24	PC(40:4)	838.6	0.322	0.077	1.1%	0.230	0.087	1.0%	-28.7%	
25	PC(40:5)	836.6	0.328	0.072	1.1%	0.218	0.102	0.9%	-33.5%	
26	PC(40:6)	834.6	0.714	0.176	2.3%	0.421	0.223	1.8%	-41.0%	*
27	PC(40:7)	832.6	0.157	0.042	0.5%	0.115	0.043	0.5%	-26.8%	
28	PC(40:8)	830.6	0.118	0.026	0.4%	0.137	0.019	0.6%	15.7%	
29	PC(42:10)	854.6	0.048	0.012	0.2%	0.051	0.012	0.2%	5.7%	
30	PC(42:11)	852.5	0.016	0.005	0.1%	0.023	0.006	0.1%	42.2%	
31	PC(42:2)	870.7	0.174	0.051	0.6%	0.150	0.041	0.6%	-13.6%	
32	PC(42:3)	868.7	0.019	0.006	0.1%	0.017	0.007	0.1%	-11.8%	
33	PC(42:4)	866.7	0.054	0.014	0.2%	0.046	0.015	0.2%	-14.8%	
34	PC(42:5)	864.6	0.057	0.013	0.2%	0.036	0.019	0.2%	-36.4%	*
35	PC(42:6)	862.6	0.033	0.009	0.1%	0.021	0.009	0.1%	-37.1%	
36	PC(42:7)	860.6	0.070	0.019	0.2%	0.043	0.021	0.2%	-38.6%	
37	PC(42:8)	858.6	0.114	0.037	0.4%	0.136	0.024	0.6%	19.1%	
38	PC(42:9)	856.6	0.083	0.022	0.3%	0.090	0.018	0.4%	8.1%	
39	PC(44:10)	882.6	0.028	0.008	0.1%	0.020	0.008	0.1%	-29.5%	
40	PC(44:11)	880.6	0.026	0.009	0.1%	0.035	0.009	0.1%	35.6%	
41	PC(44:12)	878.6	0.039	0.013	0.1%	0.029	0.013	0.1%	-26.9%	
42	PC(44:2)	898.7	0.039	0.013	0.1%	0.021	0.015	0.1%	-46.8%	*
43	PC(44:4)	894.7	0.042	0.012	0.1%	0.040	0.014	0.2%	-5.4%	
44	PC(44:5)	892.7	0.045	0.013	0.1%	0.056	0.014	0.2%	25.0%	
45	PC(44:6)	890.7	0.012	0.004	0.0%	0.010	0.004	0.0%	-18.8%	
46	PC(44:8)	886.6	0.031	0.008	0.1%	0.031	0.009	0.1%	0.0%	
47	PC(44:9)	884.6	0.039	0.012	0.1%	0.029	0.013	0.1%	-25.0%	
		TOTAL PC	30.430			23.742				

F. Lysophosphatydilcholine (LPC)

N	Name	$[M+H]^+$	r-sham (n=5) Mean	r-sham (n=5) SD	r-sham (n=5) % of total	r-mTBI (n = 5) Mean	r-mTBI (n = 5) SD	r-mTBI (n = 5) % of total	% change	Sign. change
1	LPC(16:1)	494.3	0.010	0.003	0.5%	0.015	0.004	0.6%	58.6%	
2	LPC(16:0)	496.3	0.717	0.200	39.1%	1.222	0.244	51.0%	70.4%	*
3	LPC(18:2)	520.3	0.018	0.001	1.0%	0.017	0.004	0.7%	-5.0%	
4	LPC(18:1)	522.4	0.309	0.089	16.8%	0.336	0.022	14.0%	8.9%	
5	LPC(18:0)	524.4	0.652	0.115	35.5%	0.738	0.012	30.8%	13.3%	
6	LPC(20:5)	542.3	0.012	0.002	0.6%	0.007	0.002	0.3%	-44.0%	*
7	LPC(20:1)	550.4	0.042	0.011	2.3%	0.026	0.002	1.1%	-38.4%	*
8	LPC(20:0)	552.4	0.049	0.013	2.6%	0.020	0.002	0.8%	-58.4%	*
9	LPC(22:6)	568.3	0.019	0.005	1.0%	0.011	0.003	0.5%	-41.7%	*
10	LPC(22:5)	570.4	0.009	0.003	0.5%	0.003	0.002	0.1%	-64.2%	**
		TOTAL LPC	1.836			2.396				**

G. Phosphatydilethanolamine (PE)

N	Name	[M-H]-	r-sham (n=5) Mean	SD	% of total	r-mTBI (n = 5) Mean	SD	% of total	% change	sign. change
1	PE(30:0)	662.5	0.024	0.006	0.2%	0.019	0.005	0.2%	-19.7%	
2	PE(30:1)	660.5	0.023	0.005	0.2%	0.022	0.004	0.2%	-7.2%	
3	PE(32:0)	690.5	0.036	0.009	0.2%	0.027	0.006	0.2%	-23.2%	
4	PE(32:1)	688.5	0.050	0.013	0.3%	0.050	0.013	0.4%	0.0%	
5	PE(32:2)	686.5	0.031	0.006	0.2%	0.036	0.006	0.3%	14.6%	
6	PE(34:0)	718.5	0.048	0.013	0.3%	0.038	0.008	0.3%	-21.9%	
7	PE(34:1)	716.5	0.734	0.201	4.9%	0.504	0.177	4.3%	-31.3%	
8	PE(34:2)	714.5	0.297	0.048	2.0%	0.280	0.067	2.4%	-5.9%	
9	PE(34:3)	712.5	0.129	0.029	0.9%	0.115	0.029	1.0%	-10.7%	
10	PE(34:4)	710.5	0.084	0.023	0.6%	0.065	0.019	0.6%	-22.4%	
11	PE(36:0)	746.6	0.334	0.084	2.2%	0.450	0.135	3.8%	34.9%	
12	PE(36:1)	744.6	1.732	0.386	11.6%	1.095	0.455	9.3%	-36.8%	*
13	PE(36:2)	742.5	0.859	0.199	5.8%	0.505	0.145	4.3%	-41.2%	*
14	PE(36:3)	740.5	0.194	0.040	1.3%	0.171	0.034	1.5%	-12.1%	
15	PE(36:4)	738.5	0.327	0.060	2.2%	0.303	0.075	2.6%	-7.4%	
16	PE(36:5)	736.5	0.159	0.038	1.1%	0.145	0.038	1.2%	-8.5%	
17	PE(36:6)	734.5	0.075	0.015	0.5%	0.062	0.017	0.5%	-17.0%	
18	PE(38:0)	774.6	1.523	0.343	10.2%	1.273	0.489	10.9%	-16.4%	
19	PE(38:1)	772.6	0.843	0.170	5.7%	0.829	0.320	7.1%	-1.6%	
20	PE(38:2)	770.6	0.437	0.084	2.9%	0.275	0.075	2.3%	-37.0%	*
21	PE(38:3)	768.6	0.306	0.053	2.1%	0.227	0.067	1.9%	-25.9%	
22	PE(38:4)	766.5	1.520	0.300	10.2%	1.152	0.363	9.8%	-24.2%	
23	PE(38:5)	764.5	0.511	0.076	3.4%	0.382	0.115	3.3%	-25.3%	
24	PE(38:6)	762.5	0.379	0.076	2.5%	0.256	0.086	2.2%	-32.5%	*
25	PE(40:2)	798.6	0.215	0.053	1.4%	0.198	0.057	1.7%	-7.9%	
26	PE(40:3)	796.6	0.239	0.050	1.6%	0.229	0.074	2.0%	-4.3%	
27	PE(40:4)	794.6	0.609	0.114	4.1%	0.404	0.127	3.4%	-33.6%	*
28	PE(40:5)	792.6	0.335	0.051	2.2%	0.211	0.072	1.8%	-37.0%	*
29	PE(40:6)	790.5	0.890	0.210	6.0%	0.556	0.187	4.7%	-37.5%	*
30	PE(40:7)	788.5	0.146	0.031	1.0%	0.109	0.032	0.9%	-25.6%	
31	PE(40:8)	786.5	0.122	0.034	0.8%	0.117	0.031	1.0%	-3.9%	
32	PE(42:10)	810.5	0.253	0.054	1.7%	0.255	0.089	2.2%	0.7%	
33	PE(42:4)	822.6	0.163	0.038	1.1%	0.158	0.053	1.4%	-3.0%	
34	PE(42:5)	820.6	0.107	0.015	0.7%	0.093	0.026	0.8%	-12.2%	
35	PE(42:6)	818.6	0.079	0.020	0.5%	0.066	0.019	0.6%	-16.5%	
36	PE(42:7)	816.6	0.067	0.020	0.4%	0.059	0.020	0.5%	-10.7%	
37	PE(42:8)	814.5	0.097	0.036	0.7%	0.090	0.031	0.8%	-8.1%	
38	PE(42:9)	812.5	0.126	0.040	0.8%	0.121	0.038	1.0%	-4.0%	
39	PE(44:10)	838.5	0.139	0.030	0.9%	0.119	0.037	1.0%	-14.4%	
40	PE(44:11)	836.5	0.144	0.033	1.0%	0.162	0.066	1.4%	12.4%	
41	PE(44:12)	834.5	0.168	0.034	1.1%	0.185	0.077	1.6%	10.3%	
42	PE(44:4)	850.6	0.078	0.022	0.5%	0.066	0.029	0.6%	-14.8%	
43	PE(44:8)	842.6	0.095	0.035	0.6%	0.074	0.030	0.6%	-22.3%	
44	PE(44:9)	840.6	0.097	0.032	0.7%	0.089	0.030	0.8%	-8.1%	
		TOTAL PE	14.825			11.644				

I. Lysophosphatydilethanolamine (LPE)

N	Name	[M-H]-	r-sham (n=5) Mean	SD	% of total	r-mTBI (n = 5) Mean	SD	% of total	% change	sign. change
1	LPE(16:0)	452.3	0.109	0.021	2.4%	0.130	0.020	3.1%	19.1%	
2	LPE(16:1)	450.3	0.034	0.009	0.7%	0.038	0.007	0.9%	13.1%	
3	LPE(18:0)	480.3	0.158	0.012	3.4%	0.186	0.038	4.5%	18.0%	
4	LPE(18:1)	478.3	0.665	0.221	14.4%	0.640	0.131	15.4%	-3.7%	
5	LPE(18:2)	476.3	0.023	0.007	0.5%	0.020	0.003	0.5%	-13.7%	
6	LPE(18:4)	472.3	0.027	0.010	0.6%	0.020	0.004	0.5%	-25.0%	
7	LPE(20:0)	508.4	0.048	0.014	1.0%	0.037	0.005	0.9%	-23.0%	
8	LPE(20:1)	506.3	1.071	0.257	23.1%	0.922	0.271	22.2%	-13.9%	
9	LPE(20:2)	504.3	0.069	0.017	1.5%	0.054	0.008	1.3%	-21.9%	
10	LPE(20:3)	502.3	0.054	0.005	1.2%	0.046	0.012	1.1%	-15.1%	
11	LPE(20:4)	500.3	0.190	0.032	4.1%	0.187	0.045	4.5%	-2.0%	
12	LPE(20:5)	498.3	0.110	0.030	2.4%	0.084	0.027	2.0%	-23.9%	
13	LPE(22:0)	536.4	0.076	0.023	1.6%	0.065	0.012	1.6%	-14.8%	
14	LPE(22:1)	534.4	0.436	0.102	9.4%	0.356	0.115	8.6%	-18.3%	
15	LPE(22:2)	532.4	0.088	0.015	1.9%	0.091	0.025	2.2%	3.0%	
16	LPE(22:3)	530.3	0.074	0.020	1.6%	0.072	0.031	1.7%	-3.1%	
17	LPE(22:4)	528.3	0.459	0.069	9.9%	0.394	0.122	9.5%	-14.2%	
18	LPE(22:5)	526.3	0.036	0.006	0.8%	0.032	0.006	0.8%	-9.4%	
19	LPE(22:6)	524.3	0.121	0.043	2.6%	0.125	0.034	3.0%	3.0%	
20	LPE(24:0)	564.4	0.058	0.020	1.3%	0.053	0.014	1.3%	-9.0%	
21	LPE(24:3)	558.4	0.059	0.010	1.3%	0.052	0.016	1.3%	-12.0%	
22	LPE(24:4)	556.4	0.364	0.071	7.9%	0.273	0.113	6.6%	-25.1%	
23	LPE(24:5)	554.3	0.054	0.009	1.2%	0.045	0.014	1.1%	-17.3%	
24	LPE(24:6)	552.3	0.160	0.033	3.4%	0.153	0.060	3.7%	-4.2%	
25	LPE(26:3)	586.4	0.088	0.019	1.9%	0.074	0.012	1.8%	-16.7%	
		TOTAL LPE	4.632			4.147			-10.5%	

J. Phosphatydilinositol (PI)

N	Name	[M-H]-	r-sham (n=5) Mean	SD	% of total	r-mTBI (n = 5) Mean	SD	% of total	% change	sign. change
1	PI(16:0-18:1)	835	0.056	0.015	2.8%	0.047	0.014	3.2%	-16.2%	
2	PI(16:0-20:4)	857	0.297	0.076	14.9%	0.229	0.061	15.7%	-22.8%	
3	PI(18:1-20:4)	883	0.188	0.050	9.4%	0.115	0.032	7.9%	-38.7%	*
4	PI(18:0-20:4)	885	1.358	0.394	68.3%	1.002	0.237	68.7%	-26.3%	
5	PI(18:0-22:6)	909	0.055	0.014	2.7%	0.040	0.010	2.7%	-27.3%	
6	PI(20:0-22:4)	913	0.036	0.009	1.8%	0.026	0.007	1.8%	-29.6%	
		TOTAL PI	1.989			1.458				

K. PE-Creamide (PE-Cer)

N	Name	[M-H]-	r-sham (n=5) Mean	SD	% of total	r-mTBI (n = 5) Mean	SD	% of total	% change	sign. change
1	PE-Cer(16:0)	659.5	0.092	0.027	13.1%	0.090	0.033	13.6%	-2.2%	
2	PE-Cer(16:1)	657.5	0.140	0.049	20.1%	0.142	0.048	21.6%	1.2%	
3	PE-Cer(18:0)	687.5	0.119	0.038	17.0%	0.115	0.048	17.4%	-3.2%	
4	PE-Cer(18:1)	685.5	0.281	0.094	40.3%	0.242	0.088	36.7%	-14.0%	
5	PE-Cer(24:0)	771.6	0.066	0.019	9.4%	0.071	0.024	10.7%	7.3%	
		Total PE-Cer	0.697			0.659				

L. Sphyngomyelin (SM)

N	Name	[M+H]+	r-sham (n=5) Mean	SD	% of total	r-mTBI (n = 5) Mean	SD	% of total	% change	sign. change
1	SM(16:1)	701.5	0.013	0.002	1.49%	0.012	0.005	1.14%	-11.8%	
2	SM(16:0)	703.6	0.232	0.030	26.05%	0.317	0.110	30.82%	36.6%	
3	SM(18:1)	729.6	0.033	0.006	3.72%	0.036	0.012	3.53%	9.8%	
4	SM(18:0)	731.6	0.208	0.042	23.34%	0.171	0.018	16.65%	-17.6%	
5	SM(22:1)	785.6	0.033	0.005	3.70%	0.043	0.016	4.15%	29.8%	
6	SM(22:0)	787.7	0.063	0.010	7.04%	0.054	0.006	5.30%	-13.1%	
7	SM(24:1)	813.7	0.241	0.037	27.10%	0.339	0.146	32.92%	40.3%	
8	SM(24:0)	815.7	0.067	0.012	7.57%	0.056	0.006	5.48%	-16.3%	
		Total SM	0.89			1.03			7.2%	

M. Dehydrosphyngomyelin (DSM)

N	Name	[M+H]+	r-sham (n=5) Mean	SD	% of total	r-mTBI (n = 5) Mean	SD	% of total	% change	sign. change
1	DSM(18:0)	733.6	0.023	0.005	27.3%	0.036	0.021	37.1%	60.1%	
2	DSM(22:0)	789.7	0.048	0.010	57.8%	0.046	0.013	46.8%	-4.3%	
3	DSM(24:0)	817.7	0.012	0.002	14.9%	0.016	0.007	16.1%	27.8%	
		Total DSM	0.08			0.10			27.9%	

N. Sulfatide (SU)

N	Name	[M-H]-	r-sham (n=5) Mean	SD	% of total	r-mTBI (n = 5) Mean	SD	% of total	% change	sign. change
1	SU(d18:1-18:0)	806							-15.0%	
2	SU(d18:1-22:0)	862							-19.3%	
3	SU(d18:1-22:0)	878							-15.5%	
4	SU(d18:1-24:1)	888							-9.6%	
5	SU(d18:1-24:0)	890							-16.9%	
6	SU(d18:1-24:1)	904							-5.0%	
7	SU(d18:1-24:0)	906							-11.7%	
		Total SU							-13.3%	
